# C,N‐Chelated Organogermanium(II) Hydride as Catalyst for Esterification of Aldehydes

**DOI:** 10.1002/chem.202501543

**Published:** 2025-07-02

**Authors:** Dominik Vítek, Jiří Tydlitát, Libor Dostál, Milan Erben, Štěpán Podzimek, Zdeňka Růžičková, Jaromír Vinklárek, Roman Jambor

**Affiliations:** ^1^ Department of General and Inorganic Chemistry University of Pardubice Pardubice 532 10 Czech Republic; ^2^ Institute of Organic Chemistry and Techology University of Pardubice Pardubice 532 10 Czech Republic; ^3^ Synpo a.s Pardubice 530 02 Czech Republic

**Keywords:** esterification, germylene, hydride, IR spectroscopy, NMR spectroscopy

## Abstract

The reactions of the monomeric C,N‐chelated organogermanium(II) hydride L(H)Ge·BH_3_ (**1**) with potassium alkoxides KOR provided potassium hydrido‐alkoxo‐germanato‐borates {K(THF)_2_[BH_3_·Ge(L)(H)(OR)]}_2_ (R = *t* Bu (**2**), C(CH_3_)_2_CH_2_CH_3_ (**3**)) as products of KO*R* addition. Compounds **2** and **3** react with benzaldehyde under formation of alkyl esters of benzoic acid along with elimination of a neutral complex L(H)Ge·BH_3_ (**1**). Thus complex **1** was tested as a useful catalyst for esterification of aldehydes by KO*^t^* Bu. The GC‐MS analysis revealed formation of *t*‐butyl esters of appropriated carboxylic acids. The mechanistic studies for the esterification of benzaldehyde with KO*^t^* Bu catalyzed by **1** were also performed either theoretically (DFT calculations), or experimentally (NMR and IR spectroscopy).

## Graphical abstract

Monomeric C,N‐chelated organogermanium(II) hydride L(H)Ge·BH_3_ (**1**) reacts with potassium alkoxides to give potassium hydrido‐alkoxo‐germanato‐borates {K(THF)_2_[BH_3_·Ge(L)(H)(OR)]}_2_ (R = *t* Bu (**2**), C(CH_3_)_2_CH_2_CH_3_ (**3**)) as products of KO*R* addition. Moreover, complex **1** was tested as useful catalyst for esterification of aldehydes by KO*^t^* Bu to yield *t*‐butyl esters of appropriated carboxylic acids.

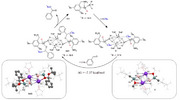

## 1 Introduction

Reactivity of tetrylene‐hydrides RE(II)*H* (E is Si, Ge, Sn, Pb) toward unsaturated bonds classifies them as a very important class of organometallic compounds, which can be even compared to transition metal hydrides.^[^ ^1^ ^]^ Studies made by groups of Power,^[^ ^2^ ^]^ Aldridge,^[^ ^3^ ^]^ Rivard,^[^ ^4^ ^]^ Wesemann,^[^ ^5^ ^]^ Jones,^[^ ^6^ ^]^ or Roesky^[^ ^7^ ^]^ nicely clarified that the hydrometallation may proceed also without the addition of catalyst. Substrate insertions into the E–H bond should be the first step of potential hydrometallation catalytic cycles. However, the recovering of the low‐valent group 14 hydrides usually remains main disadvantage, despite the progress made by above mentioned groups.^[^ ^2^, ^3^, ^4^, ^5^, ^6^, ^7^ ^]^ Nowadays, tetrylene‐hydrides RE(II)*H* are studied as active sites for catalysis. Depending on the system, these compounds are either used as isolable species, or are formed in situ from precatalysts.^[^ ^[Link]^, ^[Link]^ ^]^ The foundation of their use was laid by the group of Jones, who demonstrated that two‐coordinate tetrylenes hydrides [(N^Ar^)(H)E:] (E = Ge, Sn, N^Ar^ = [N(Ar)(SiMe_3_)], Ar = [C_6_H_2_Me{C(H)Ph_2_}_2_‐2,4,6]) may be used as efficient catalysts in the hydroboration of aldehydes or ketones RR^1^C═O by the pinacol borane (HBpin).^[^ ^8^ ^]^ Since that time, tetrylenes hydrides or their precatalysts have been tested more frequently as suitable catalysts for hydroboration, hydrosilylation, or cyanosilylation of aldehydes, ketones, pyridine, or nitriles.^[^ ^5^, ^8^, ^9^ ^]^ The conversion of CO_2_ into the methanol equivalents MeOBR_2_ (R = Bpin or Bcat) using the HBpin or HBcat was achieved either by tetrylenes hydrides precatalysts [(L´)(O*^t^* Bu)E:] (E = Ge, Sn, L´ = [N(Ar*)(Si*^i^* Pr_3_)], Ar* = [C_6_H_3_{C(H)Ph_2_}_2_‐2,4,6]) reported by Jones, Maron, and coworkers,^[^ ^10^ ^]^or by Si(II) hydride complex [(ImMe_4_)_2_SiH]I [ImMe_4_ =: C(NMe)_2_(CMe)_2_] reported by So and coworkers.^[^ ^11^ ^]^ The Power group also successfully used Sn(II) hydride precatalysts [(Ar^Me6^)(μ‐OMe)Sn:]_2_ and [(Ar^iPr4^)(μ‐OMe)Sn:]_2_ (Ar^Me6^ = Ar^Me6^ = [C_6_H_3_{C(H)Ph_2_}_2_‐2,4,6]) in dehydro‐coupling reactions of amines R_2_NH (R = H, alkyl, aryl) with HBpin.^[^ ^12^ ^]^ Finally, the Driess group opened another direction of tetrylene‐hydride catalysis when they have reported on the cooperative activation of H_2_ by a bimetallic Ni(0)/Si(II) system, and postulated active Ni(μ‐H)_2_Si structure.^[^ ^13^ ^]^ Recently, we have started with studies including N→Ge coordinated germanium hydride L(H)Ge (L is [2‐(CH_2_NEt_2_)‐4,6‐*t*Bu_2_‐C_6_H_2_]^−^) and its adducts with ZnCl_2_ and BH_3_.^[^ ^14^ ^]^ While the [L(H)Ge·ZnCl_2_]_2_ is an active catalyst in the ring‐opening polymerization of the lactide, ^[^ ^14a^ ^]^ the borane adduct L(H)Ge·BH_3_ (**1**) reacted with organolithium salts RLi (R = Ph, *t*Bu, *n*Bu) to produce lithium hydrido‐germanato‐borates {Li(THF)_2_{BH_3_[L(H)GeR]}}_2_.^[^ ^14b^ ^]^ Here we report on the reactivity studies of **1** with potassium alkoxides, and subsequent use of **1** as an unpreceded catalyst in a direct esterification of aldehydes.

## 2 Results and Discussion

**Scheme 1:**
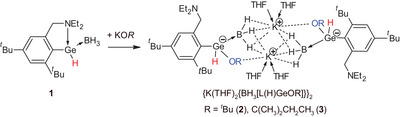
Synthesis of {K(THF)_2_[BH_3_·Ge(L)(H)(OR)]}_2_ (R = *t* Bu (**2**), C(CH_3_)_2_CH_3_CH_3_ (**3**).

The monomeric germanium hydride L(H)Ge·BH_3_ (**1**) was prepared according to our previous procedure.^[^ ^14b^ ^]^ Complex **1** was then treated with different alkali alkoxides MO*R* (M = Li, Na, K; R = *^t^* Bu, C(CH_3_)_2_CH_2_CH_3_). While no reactions were observed for M = Li or Na, reactions of potassium alkoxides KOR provided potassium hydrido‐alkoxo‐germanato‐borates {K(THF)_2_[BH_3_·Ge(L)(H)(OR)]}_2_ (R = *t* Bu(**2**), C(CH_3_)_2_CH_2_CH_3_ (**3**)) as products of KO*R* addition (Scheme 1). These reactions thus mimic the addition of organolithium salts RLi (*t* Bu, *n*Bu, Ph) into **1**.^[^ ^14b^ ^]^ Compounds **2** and **3** were characterized via NMR spectroscopy and X‐ray diffraction analysis.

The ^1^H NMR spectra of THF‐d_8_ solutions of **2** and **3** showed that the Ge*H* proton resonates at *δ* 6.35 ppm and the ^11^B{^1^H} NMR spectra of **2** and **3** revealed resonances at *δ* −37.6 (**2**) and −37.4 (**3**). The data correlate with those found for lithium germanato‐borates {Li(THF)_2_{BH_3_[L(H)GeR]}}_2_ (*δ* ^1^H ranges from 4.93 to 5.65, *δ* ^11^B ∼ −41 ppm)^[^ ^14b^ ^]^ and [{HC(C(CH_2_)NAr)CMeNAr}Ge(H)BH_3_]Li(OEt_2_)_3_ (Ar = 2,6‐*i*Pr_2_C_6_H_3_, *δ* ^1^H 6.70 ppm, *δ* ^11^B at −43.7 ppm),^[^ ^15^ ^]^ and proved the existence of negatively charged {BH_3_[L(H)GeR]}^−^ moiety. The IR spectra showed typical vibrations of both B*H* and Ge*H* bonds at *ν*_BH3_ 2343 (**2**) or 2356 (**3**) cm^−1^, and *ν*_GeH_ 1989 (**2**) or 1976 (**3**) cm^−1^.^[^ ^16^ ^]^

**Figure 1:**
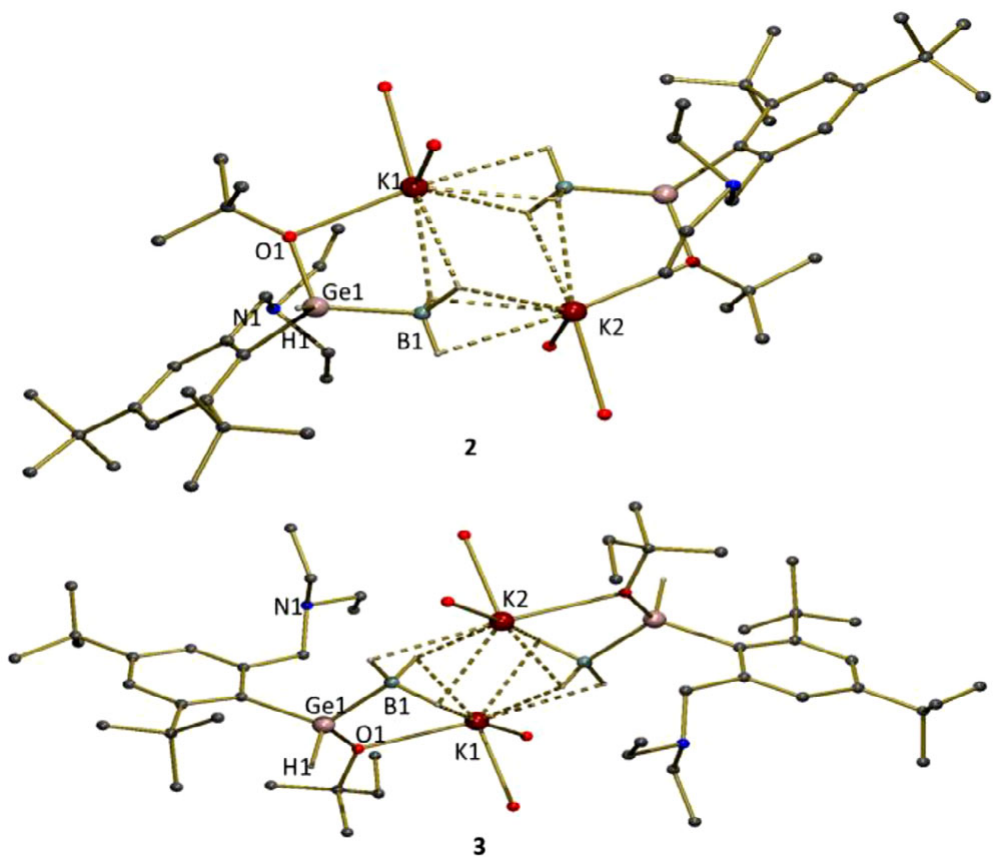
Molecular structures of **2** and **3**. Hydrogen atoms (except of the Ge*H*) and coordinated molecules of THF are omitted. Selected bond distances (Å) and angels (°): For **2**: Ge1─O1 1.8428(17), Ge1─B1 2.053(3), K1─O1 2.700(2), Ge1─H1 1.49(4), O1─Ge1─C1 105.99(9), O1─Ge1─B1 105.36(10). For **3**: Ge1─O1 1.845(2), Ge1─B1 2.050(4), K1─O1 2.706(2), Ge1─H1 1.48(4), O1─Ge1─C1 106.70(11), O1─Ge1─B1 106.04(13).

Compounds **2** and **3** crystallize as discrete centrosymmetric dimers (Figure 1, for details see Supporting Informtion).^[^ ^17^ ^]^ The {BH_3_[L(H)GeR]}^−^ ligand binds one potassium K1 through the O1 atom (2.700(2) Å in **2**, 2.706(2) Å in **3**), while the hydrogen atoms of borane coordinate second potassium K2. In addition, two hydrogens also coordinate the K1 ion and thus the borane group forms bridge between both potassium ions forming diamond‐shaped K_2_(BH_3_)_2_ core. The potassium ions are also coordinated by two THF molecules defining the potassium ions pseudo‐eight coordinate. Similar arrangement was observed in potassium salts of amido‐diphenylphosphine‐borane ligands,^[^ ^18^ ^]^ and thio‐substituted phosphido‐borane ligands.^[^ ^19^ ^]^ The Ge atoms have the tetrahedral arrangement. The Ge–B bond distances (2.053(3) Å in **2**, 2.050(4) Å in **3**) suggest strong Ge→B interaction (Σ_cov_Ge,B = 2.06 Å).^[^ ^20^ ^]^ The Ge–O bond distances (1.8428(2) Å in **2**, 1.845(2) Å in **3**) are closed to the sum of the covalent radii of the parent atoms (Σ_cov_Ge,O = 1.84 Å).^[^ ^20^ ^]^

Thus, it seems that reactivity of **1** toward nucleophiles (Nu^−^) has a general character. The Nu^−^ group may coordinate the Ge(II) atom and convert a neutral L(H)Ge(II) moiety into the organogermanato anions [L(H)Ge(II)R]^−^ (R is Nu^−^). This process is accompanied with the decoordination of the Et_2_N arm of L. The flexibility and stability of **1** contrast with a related germanium hydride [{HC(CMeNAr)_2_}GeH(BH_3_)] (Ar = 2,6‐*i*Pr_2_C_6_H_3_) that provided [{HC(C(CH_2_)NAr)CMeNAr}Ge(H)BH_3_]Li(OEt_2_)_3_ along with the elimination of *^t^* BuH.^[^ ^15^ ^]^

**Scheme 2:**
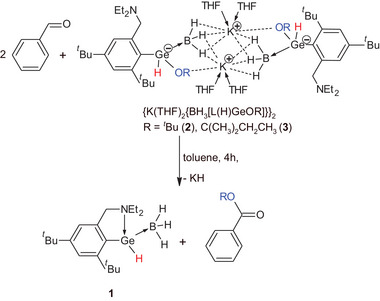
Stoichiometric reaction of **2** and **3** with benzaldehyde.

Since these compounds **2** and **3** contain both B─H and Ge─H bonds, their ability to reduce C═O bond was assessed by the stoichiometric reaction with benzaldehyde (Scheme 2, for experimental details see Supporting Information), similarly to their lithium analogues {Li(THF)_2_{BH_3_[L(H)GeR]}}_2_.^[^ ^14b^ ^]^ Interestingly, the GC‐MS analysis of a reaction mixture showed formation of appropriate alkyl esters ROC(O)Ph (R = *^t^* Bu, C(CH_3_)_2_CH_2_CH_3_) with conversions up to 95% (Scheme 2, Figures S9 and S10 in Supporting Information). In addition, monitoring of stoichiometric reactions in C_6_D_6_ revealed signal of the Ge*H* proton at *δ* 6.54 ppm, characteristic for L(H)Ge·BH_3_ (**1**), and suggested a recovery of neutral germanium hydride **1** during this reaction (Figure S11 in Supporting Information).

The formation of **1** in the reaction mixture also suggests, that Ge*OR* group of the starting **2** or **3** is transferred to benzaldehyde and new C*OR* bond is formed to provide parent esters of benzoic acids, as detected by GC‐MS analysis. Thus, compound **1** appears as a starting material for synthesis of **2**/**3**, as well as the product of abovementioned reactions of **2/3** with benzaldehyde.

**Scheme 3:**
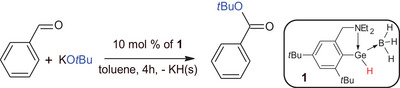
Esterification reaction of benzaldehyde with KO*^t^* Bu catalyzed by **1**.

Therefore, **1** could be a useful catalyst for esterification of benzaldehyde. Thus, the reaction of benzaldehyde with KO*^t^* Bu in the presence of **1** as the catalyst (10 mol %) was done under same conditions (Scheme 3). Once more, the GC‐MS analysis revealed formation of *t*‐butyl ester of benzoic acid as the major product with conversion up to 95% (Figure S12 in Supporting Information). To see the effect of **1**, a blind experiment between benzaldehyde and KO*^t^* Bu in an absence **1** was carried out as well. The GC‐MS analysis revealed presence of benzyl ester of benzoic acid as the major product (conversion ∼ 90%, Figure S15A in Supporting Information), typical for Tishchenko reaction. We have also done blind experiment between benzaldehyde and KO*^t^* Bu in the presence of BH_3_ as the catalyst (10 mol %) to see effect of the Ge atom. The GC‐MS analysis revealed presence of *t*‐butyl ester of benzoic acid, but benzyl alcohol was detected as the major product (conversion ∼ 60%, Figure S15B in Supporting Information).

Therefore, the presence of **1** shifts progress of the reaction toward alkyl esters of benzoic acid.

It should also be noted that the decrease of catalytic loading of **1** to 1% provided mixture of *t*‐butyl ester of benzoic acid (∼70%) and benzyl alcohol (∼ 30%, Figure S14 in Supporting Information). The effect of cations M (M = Li, Na, K) in MO*^t^* Bu was also tested. Therefore, reactions of benzaldehyde with MO*^t^* Bu (M = Li or Na) in the presence of **1** as the catalyst (10 mol %) were done, but no *t*‐butyl ester of benzoic acid was detected by GC‐MS analysis (Figure S13). However, this observation, fits well with low reactivity of **1** toward MO*R* for M = Li and Na. Therefore, **1** cannot be converted into the appropriate hydrido‐alkoxo‐germanato‐borate.

**Figure 2:**
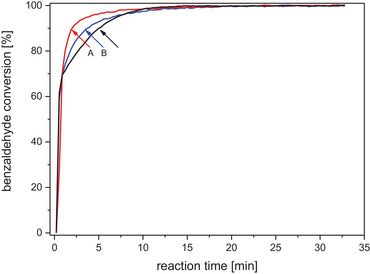
The courses of stoichiometric (**A**, **B**) and catalytic (**C**) reactions monitored by time‐resolved FTIR spectroscopy. Experimental conditions: **A**) benzaldehyde + KO*^t^* Bu + **1**, **B**) benzaldehyde + **2**, **C**) benzaldehyde + KO*^t^* Bu + 10 mol% of **1**.

The courses of studied reactions were also monitored by IR spectroscopy using ATR probe following the changes of the band area corresponding to C═O stretching mode of benzaldehyde (Figure 2). We have observed, that stoichiometric and catalytic (10 mol. % of **1**) reactions proceed very fast and starting compounds are converted into respective ester within 15 minutes. We note that using of catalytic amount of **1** lead to a slight decrease in the reaction rate when 70% of conversion is achieved. Thus we propose, that in the catalytic reaction catalyst **1** reacts with KO*^t^* Bu to provide **2** that may react with the benzaldehyde as a rate determining step. The regeneration of **1** is the final step and it can repeatedly react with KO*^t^* Bu to give **2**. A series of reactions performed at various stoichiometry proved that conversion of benzaldehyde corresponds to the amount of KO*^t^* Bu used in the reaction, see Figure S16 in Supporting Information. This again confirms, that **1** reacts with KO*^t^* Bu to provide **2**, and this step is then followed by the reaction with benzaldehyde.

**Scheme 4:**
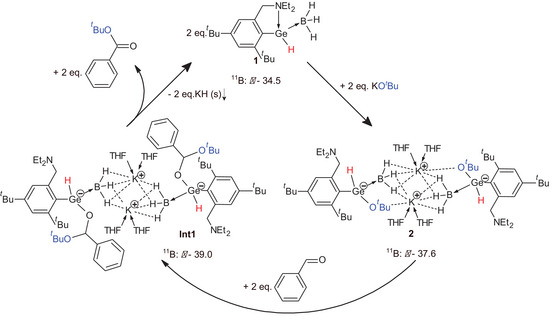
Catalytic cycle for the esterification of benzaldehyde with KO*^t^* Bu catalyzed by **1** proposed by DFT calculations (optimalization of **2**, **Int1,** and Δ*G*) in the combination with the experimental studies (NMR and IR spectroscopy).

**Figure 3:**
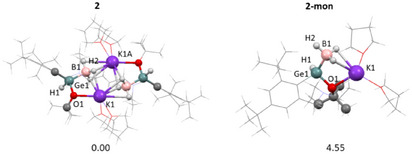
Optimized geometries of **2** and **2‐mon** and relative Gibbs free energies (in kcal mol^−1^; calculated in *n*‐hexane at the M06/cc‐pVTZ(‐PP) level).

To explain the rapid consumption of the C═O bond of the benzaldehyde together with the formation of new C*O^t^* Bu bond in *t*‐butyl ester of benzoic acid, we tentatively propose, that reaction might proceed via a prereactive intermediate **Int1**, as the addition product of the GeO*^t^* Bu bond of **2** to the C═O bond of the benzaldehyde. Finally, **Int1** with new C*O^t^* Bu bond may decompose to **1** and *t*‐butyl ester of benzoic acid along with elimination of KH (Scheme 4). To support this proposed mechanism, DFT computational studies together with additional ^11^B NMR experiments were done. DFT calculations were performed to gain a better understanding of the molecular structure of **2** and suggested **Int1**. The geometries of both complexes were fully optimized at the M06/cc‐pVDZ(‐PP)^[^ ^21^ ^]^ level of theory, which was chosen on the basis of our previous studies of lithium organogermanato compounds {Li(THF)_2_{BH_3_[L(H)GeR]}}_2_ (R is Ph, *t*Bu, *n*Bu),^[^ ^14b^ ^]^ or of the transition metal complexes of the type L(H)Ge·TM(CO)_5_.^[^ ^22^ ^]^ We have previously demonstrated, that dimeric structures of the lithium organogermanato compounds {Li(THF)_2_{BH_3_[L(H)GeR]}}_2_ (R is Ph, *t*Bu,*n*Bu),^[^ ^14b^ ^]^ analogues of **2**, are more stable than its monomers Li(THF)_2_{BH_3_[L(H)GeR]}. In the case of studied complex **2** and plausible corresponding monomer (**2‐mon**) the DFT calculations confirmed stabilization of dimeric structure by 4.55 kcal/mol (see Figure 3).

**Figure 4:**
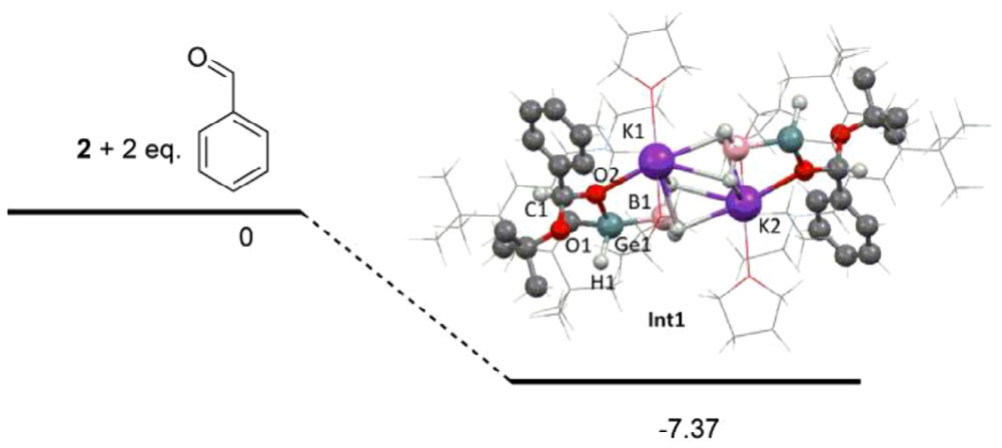
Energetical profile for the reaction of **2** with 2 eq. of benzaldehyde to give **Int1** (in kcal mol^−1^; calculated in *n*‐hexane at the M06/cc‐pVTZ(‐PP) level).

The agreement between the experimental X‐ray diffraction data of **2** and the calculated structural parameters was deemed satisfactory (Figure 3). The structure is very similar, showing a diamond‐shaped K_2_(BH_3_)_2_ core and four‐coordinate Ge center with a distorted tetrahedral geometry with bond lengths Ge─O 1.889 Å; Ge─C 2.029 Å; Ge─B 2.073 Å. The structure of **Int1** closely resembles that of **2** and **Int1** remains as hydrido‐alkoxo‐germanatoborate {K(THF)_2_[BH_3_·Ge(L)(H)(OR)]}_2_ (R = C(O*t*Bu)(H)Ph) having four‐coordinate Ge center with Ge→B interaction (2.073 Å) (Figure 4). In **Int1**, new Ph(H)C‐*O^t^* Bu (C1─O1 = 1.416 Å) and Ge─O─C(H)Ph bonds (Ge1─O2 = 1.912 Å) are formed. Oxygen O2 of Ge─O─C(H)Ph bond also interacts with potassium cation (K1─O2 2.665 Å). In addition, according to the DFT calculations, reaction of **2** with two eq. of benzaldehyde might proceed via a prereactive intermediate **Int1** (Figure 4). The original GeO*^t^* Bu bond of **2** is cleaved in **Int1** (Ge1─O2 = 3.260 Å). Similarly, former C═O bond of benzaldehyde was transformed during this addition to C1─O2 bond with bond length of 1.376 Å in **Int1**. In addition, the whole process is a thermodynamically favored with Δ*G* = −7.37 kcal/mol (Figure 4).

Since the proposed mechanism involve the elimination of solid KH, DFT calculations could not provide valid information about the last step of suggested mechanism. Therefore, we have done both stoichiometric (Scheme 2) and catalytic (Scheme 3) reactions at −50°C in THF to slow down the reaction rate, and we monitored reactions by ^11^B{^1^H} NMR spectroscopy. After 10 minutes, the stoichiometric reaction revealed signals at *δ* −37.6 of starting **2** together with a new signal at *δ* −38.6 (Figure S17 in Supporting Information). This new signal corroborates formation of new {BH_3_[L(H)GeR]}^−^ moiety (**Int1**) in reaction. In contrast after 60 minutes, the ^11^B NMR spectrum showed signals at *δ* −38.6 of **Int1** and at *δ *−34.5 ppm of **1**, while the signal of starting **2** disappeared (Figure S18 in Supporting Information). Similarly, catalytic reaction reveled signals at *δ *−34.5 (**1**), −38 (**2**), and −38.6 ppm (major signal of **Int1**) after 10 minutes (Figure S19 in Supporting Information), and at *δ *−35.5 (**1**) and −39.6 ppm (major signal of **Int1**) after 60 minutes (Figure S20 in Supporting Information). These data again corroborate reaction of **1** with KO*^t^* Bu under formation of **2** and its immediate reaction with benzaldehyde probably via hydrido‐alkoxo‐germanato‐borate **Int1** (supported by DFT and experimental studies). Degradation of **Int1** may provide **1** and *t*‐butyl ester of benzoic acid along with elimination of KH (Scheme 4). As the reaction is heterogenous (see Figure S26 in Supporting Information), the solid material was separated and characterized by Raman spectroscopy as KH (see Figure S27 in Supporting Information). Therefore, all the data support proposed mechanism depicted in Scheme 4.

**Scheme 5:**
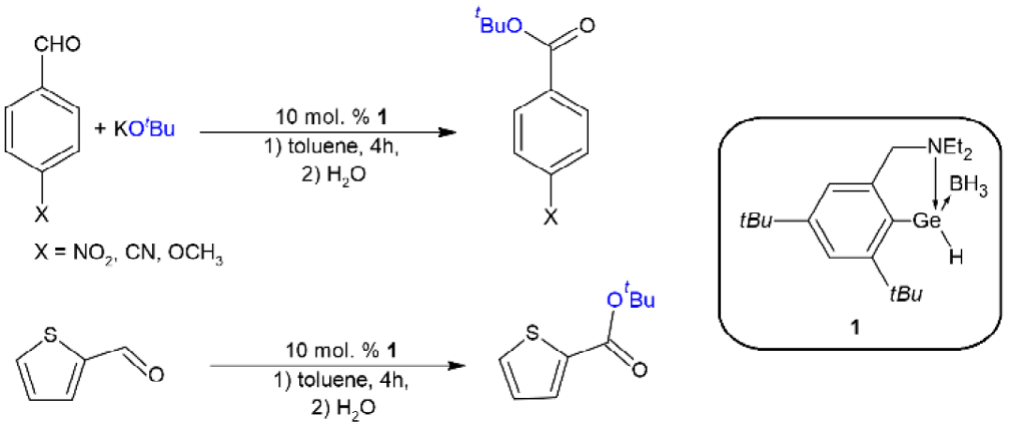
Esterification of substituted aldehydes by KO*^t^* Bu catalyzed by **1**.

We have also done some preliminary tests for the scope of substrates. Therefore, reactions of substituted aldehydes with KO*^t^* Bu in the presence of **1** as the catalyst (10 mol %) were done under same conditions (Scheme 5). The GC‐MS analysis of the reaction mixtures revealed nearly quantitative formation of appropriate *t*‐butyl esters with conversion up to 95% (Figures S21–S24 in Supporting Information).

Finally, we have also run the esterification reaction of 4‐CN benzaldehyde as the up‐scale experiment (200 mg) and corresponding *t*‐butyl ester of 4‐CN‐benzoic acid was isolated and characterized by ^1^H NMR spectroscopy (Figure S25 in Supporting Information)

## 3 Conclusion

In conclusion, we have reported on the synthesis of potassium hydrido‐alkoxo‐germanato‐borates K(THF)_2_[BH_3_·Ge(L)(H)(OR)]}_2_ (R = *t* Bu(**2**), C(CH_3_)_2_CH_2_CH_3_ (**3**)) as products of KO*R* addition to neutral L(H)Ge·BH_3_ (**1**). Both compounds **2** and **3** react with benzylaldehyde to provide appropriate alkyl esters of benzoic acid and eliminates starting **1**. This demonstrates possible application of **1** as a catalyst for esterification of benzaldehyde by KO*^t^* Bu. This unique application of tetrylene hydrides was not reported up to date. The possible application of **1** as a catalyst for the esterification of several substrates was demonstrated and it was also clearly proven, that the presence of 10 mol. % of **1** shifts progress of the reaction toward alkyl esters of benzoic acid from the Tishchenko reaction. Mechanistic studies also support formation of **2** by the reaction of **1** and KO*^t^* Bu in the catalytic cycle. Reaction of **2** with the C═O bond of the benzaldehyde may process as addition reaction of the GeO*^t^* Bu bond of **2** to the C═O bond of the benzaldehyde via a prereactive intermediate **Int1**. The addition reactions of the GeO*R* bond in N‐coordinated germylenes are thus of current interest.

## Conflict of Interest

The authors declare no conflict of interest.

## Supporting information



Supporting Information

Supporting Information

## Data Availability

The data that support the findings of this study are openly available in figshare at 10.6084/m9.figshare.28853477, reference number 0.
