# Supplementary material for: C,N‐Chelated Organogermanium(II) Hydride as Catalyst for Esterification of Aldehydes
Source: Chemistry. 2025 Jul 2;31(40):e202501543. doi: 10.1002/chem.202501543 (PMC12272026; doi:10.1002/chem.202501543)
Supplement: Supplementary file 1 — Supporting Information [file CHEM-31-e202501543-s002.pdf]

Supporting Information

C,N-Chelated Organogermanium(II) Hydride as Catalyst for Esterification of Aldehydes

Dominik Vítek,[a] Jiří Tydlitát,[b] Libor Dostál,[a] Milan Erben,[a] Štěpán Podzimek,[c] Zdeňka Růžičková,[a] Jaromír Vinklárek,[a] Roman Jambor[a]*

Content:

Experimental part	p.2 – p.6
Figures S1 – S26	p.7 – p. 34
Table S1. Crystallographic parameters for **2** and **3**	p.35
Results of DFT calculations	p.36 – p.46
References	p.48

General considerations

Solvents were dried by standard methods and distilled prior to use. The deuterated solvents were purchased from GenChem and dried over 4 Å molecular sieves. All moisture and air sensitive reactions were carried out under an argon atmosphere by using standard Schlenk techniques. ^{1}H, $^{13}C\{^{1}H\}$ and $^{11}B\{^{1}H\}$ NMR spectra were recorded on a Bruker Avance 400 and 500 MHz NMR spectrometer at 298 K. The ^{1}H and $^{13}C\{^{1}H\}$ NMR spectra were referenced internally to residual proton-solvent and solvent resonances, respectively, and are reported relative to Me_4Si (δ = 0 ppm). Time-resolved infrared spectra were obtained with Si-ATR dip probe (Art Photonics) on a Nicolet iS50 FT-IR spectrometer in the range 600-3500 cm^{-1} (resolution 4 cm^{-1}, sampling interval of 12.56 sec). The melting or decomposition temperature of compounds was determined on a Stuart MP3 thermometer in a glass capillary. The mass spectra of toluene solutions of prepared esters were measured on GC/MS configuration comprised of an Agilent Technologies-6890N gas chromatograph (HP-5MS column, length 30 m, I.D. 0.25 mm, film 0.25 lm) with He gas as mobile phase, equipped with a 5973 Network MS detector (EI 70 eV, mass range 33-550 Da). Raman spectra of solid samples sealed in NMR tubes were measured on iS50 Raman module (excitation laser 1064 nm, resolution 8 cm^{-1}). Elemental analyses were performed with a LECO-CHNS-932 analyzer.

Starting materials

1,7M toluene solution of $KOC(CH_3)_2CH_2CH_3$, 1M THF solution of KO^tBu and all aldehydes were purchased on Sigma-Aldrich. Complex **1** was prepared according to literature procedure. [14b]

Synthesis of $\{K(THF)_2[BH_3{\cdot}Ge(L)(H)(O^tBu)]\}_2$ (**2**).

A THF solution (10 ml) of $L(H)Ge{\cdot}BH_3$ (**1**) (139.1 mg, 0.38 mmol) was cooled to -50°C. Subsequently, 1M THF solution (0.38 ml, 0.38 mmol) of KO^tBu was added dropwise into the solution, the reaction mixture was slowly heated up to room temperature and stirred for 1.5h. Organic solvent was evaporated and resulting white powder of **2** dried under vacuum. Crystals suitable for XRD analysis were obtained from a concentrated THF solution of **2** at -20°C.

Characterization of ***2***: Yield: 0.18g, (99%); m.p. 94.1 °C-decomp.; Elemental analysis calcd. for $C_{62}H_{122}B_2Ge_2K_2N_2O_6$ (Mw = 1236.74): Calculated: C 60.2, H 9.9, N 2.3, found C 60.9, H 10.4, N 2.9; ^{1}H NMR (d_8-THF, 500 MHz): δ 1.06 (m, 6H, $CH_2C\mathit{H}_3$), 1.06 (s, 9H, $C(C\mathit{H}_3)_3$), 1.30 (s, 9H, $C(C\mathit{H}_3)_3$), 1.48 (s, 9H, $C(C\mathit{H}_3)_3$), 2.48 (m, 2H, $C\mathit{H}_2CH_3$), 2.60 (m, 2H, $C\mathit{H}_2CH_3$),

4.21 (s, 2H, CH_2N), 6.35 (br, 1H, Ge*H*), 7.22 (s, 1H, Ar*H*), 7.70 (s, 1H, Ar*H*). $^{13}C\{^1H\}$ NMR (d_8-THF, 125 MHz): δ 12.7, 25.4, 31.0, 31.1, 32.4, 34.2, 37.7, 47.5, 58.6, 70.5, 119.2, 121.1, 144.2, 147.2, 147,4, 155.9. $^{11}B\{^1H\}$ NMR (d_8-THF, 160 MHz): δ -37.63. IR (diamond ATR, cm^{-1}): 2343s, 2288s, 2260s (ν_{BH}), 1989m-br (ν_{GeH}).

Synthesis of $\{K(THF)_2[BH_3{\cdot}Ge(L)(H)(OC(CH_3)_2CH_2CH_3)]\}_2$ (**3**).

A THF solution (10 ml) L(H)Ge·BH_3 (**1**) (110 mg, 0.35 mmol) was cooled to -50°C. Subsequently, 1.7M toluene solution (0.20 ml, 0.35 mmol) of $KOC(CH_3)_2CH_2CH_3$ was added dropwise into the solution, the reaction mixture was slowly heated up to room temperature and stirred for 1.5h. Organic solvent was evaporated and resulting white powder of **2** dried under vacuum. Crystals suitable for XRD analysis were obtained from a concentrated THF solution of **3** at -20°C.

*Characterization of **3***: Yield: 0.128g, (99%); m.p. 90.1°C-decomp.; Elemental analysis calcd. for $C_{64}H_{126}B_2Ge_2K_2N_2O_6$ (Mw = 1264.78): Calculated: C 60.8, H 10.0, N 2.2, found C 61.5, H 10.5, N 2.9; ^1H NMR (d_8-THF, 500 MHz): δ 0.99 (m, 6H, CCH_3), 1.07 (m, 3H, CCH_3), 1.10 (s, 2H, OCH_2), 1.31 (s, 9H, C(CH_3)$_3$), 1.50 (s, 9H, C(CH_3)$_3$), 2.49 (m, 2H, CH_2CH_3), 2.60 (m, 2H, CH_2CH_3), 4.21 (s, 2H, CH_2N), 6.35 (br, 1H, Ge*H*), 7.23 (s, 1H, Ar*H*), 7.73 (s, 1H, Ar*H*). $^{13}C\{^1H\}$ NMR (d_8-THF 125K): δ 12.9, 27.9, 28.1, 31.1, 32.5, 34.3, 36.7, 37.7, 47.5, 58.8, 72.9, 119.3, 121.3, 143.7, 147.4, 147.5, 152.9. $^{11}B\{^1H\}$ NMR (d_8-THF, 160 MHz): δ -37.43. IR (diamond ATR, cm^{-1}): 2356s, 2263m (ν_{BH}), 1976m-br (ν_{GeH}).

General procedure for stoichiometric reactions of $\{K(THF)_2[BH_3{\cdot}Ge(L)(H)(OR)]\}_2$ *with benzaldehyde.*

A toluene solution (5 ml) of benzaldehyde (41 µl, 0.41 mmol) was cooled to -50°C and added to a toluene solution (5 ml) of **2** (196 mg, 0.41 mmol). The resulting mixture was stirred for 24h. Organic solvent was evaporated and resulting residue dried under vacuum. Product was then analyzed by ^1H NMR spectroscopy. Furthermore, the product was subsequently hydrolyzed, and its toluene solution analyzed by GC/MS.

*Catalytic study. Typical procedure for esterification of benzaldehyde catalyzed by **1**.*

Benzaldehyde (254 µl, 2.49 mmol) was added into a toluene solution (5 ml) of L(H)Ge·BH_3 (**1**) (90 mg. 0.249 mmol) and subsequently 1M THF solution (2.49 ml, 2.49 mmol) of KO^{t}Bu

was added into a reaction mixture. The solution was stirred for 4h, organic solvent was evaporated and resulting residue dried under vacuum. Product was subsequently hydrolyzed, and its toluene solution analyzed by GC/MS.

Identical reaction was also carried out without a presence of a catalyst **1** to confirm its necessary presence in a reaction mixture to mainly obtain a product of esterification.

*FTIR study. Typical procedure for esterification of benzaldehyde catalyzed by **1**.*

Si-ATR probe was immersed into stirred THF solution (5 ml) of benzaldehyde and catalyst **1**. Subsequently, a THF solution of KO^{t}Bu (1 M) was added at once and time-dependent changes of band area (1724-1694 cm^{-1}, C=O stretching mode) were analyzed. Obtained data showed varying conversion of benzaldehyde into *tert*-butoxyester of benzoic acid accordingly to stoichiometry used. Reaction stoichiometry was carried out in three different ratios of benzaldehyde : KO^{t}Bu : **1** (1:1:1, 1:0.5:0.5, and 1:0.1:0.1, respectively), see Figure S16. The consumption of benzaldehyde during of stoichiometric reaction with **2** and under catalytic conditions (10 mol.% of **1**) was followed analogously.

^{11}B NMR catalytic study.

Benzaldehyde (61 µl, 0.6 mmol) was added into a THF solution (5 ml) of **2** (283 mg, 0.6 mmol) cooled to -50°C. After 10 minutes, a sample was taken and ^{11}B NMR was measured, showing a peak of a new borate intermediate atop of an original signal from **2**. The reaction mixture was allowed to stir for additional 1h at low temperature. Boron NMR spectrum of this sample showed a higher conversion of the original complex **2** into the new borate intermediate. Lastly, the reaction mixture was heated up to the room temperature and stirred for 4h. NMR analysis of the resulting mixture showed only a presence of a new borate intermediate and a signal of catalyst **1**.

Benzaldehyde (14 µl, 0.14 mmol) was added into a THF solution (5 ml) of **1** (52 mg, 0.14 mmol) cooled to -50°C. Subsequently, a 1M THF solution (144 µl, 0.14 mmol) of KO^{t}Bu was added into the reaction mixture. After 10 minutes, a sample was taken and ^{11}B NMR was measured, showing a resonances of **1**, **2** and a newly formed borate intermediate that corresponds with those detected in a reaction mixture discussed above. The reaction mixture was allowed to stir for an additional hour at low temperature. ^{11}B NMR spectrum displayed a peak of catalyst **1** and newly formed borate intermediate.

*Catalytic study. Typical procedure for esterification of 4-substituted aromatic aldehydes catalyzed by **1**.*

Corresponding aromatic aldehyde was added into a toluene solution (5 ml) of **1** (10 mol. %) and subsequently, according to stoichiometry, a THF solution of KO^{t}Bu was added into a reaction mixture. The solution was stirred for 4h, organic solvent was evaporated and resulting residue dried under vacuum. Product was subsequently hydrolyzed, and its toluene solution analyzed by GC/MS.

Large scale experiment of esterification of 4-cyanobenzaldehyde.

4-cyanobenzaldehyde (200 mg, 1.53 mmol) was added into a toluene solution (5 ml) of **1** (55 mg, 0.153 mmol) and subsequently 1M THF solution (1.53 ml, 1.53 mmol) of KO^{t}Bu was added into a reaction mixture. The solution was stirred for 4h, organic solvent was evaporated and resulting residue dried under vacuum. Resulting product was then analyzed by ^{1}H NMR spectroscopy. *Characterization of t-butyl ester of 4-cyanophenyl carboxylic acid*: Yield: 210 mg, (67%); Elemental analysis calcd. for $C_{12}H_{13}NO_2$ (Mw = 203.23): Calculated: C 70.9, H 6.5, N 6.9, found C 69.9, H 6.4, N 6.7; ^{1}H NMR (C_6D_6, 500 MHz): δ 1.36 (s, 9H, C*CH_3*), 6.89 (d, 2H, Ar*H*), 7.69 (d, 2H, Ar*H*),

Crystallography.

Full-sets of diffraction data for **2**, **3** were collected at 150(2)K with a Bruker D8-Venture diffractometer equipped with Cu (Cu/Kα radiation; λ = 1.54178 Å) or Mo (Mo/Kα radiation; λ = 0.71073 Å) microfocus X-ray (IμS) sources, Photon CMOS detector and Oxford Cryosystems cooling device was used for data collection. The frames were integrated with the Bruker SAINT software package using a narrow-frame algorithm. Data were corrected for absorption effects using the Multi-Scan method (SADABS). Obtained data were treated by XT-version 2018/1 and SHELXL-2017/1 software implemented in APEX3 v2016.5-0 (Bruker AXS) system. Hydrogen atoms were mostly localized on a difference Fourier map, however to ensure uniformity of treatment of crystal, all hydrogen atoms were recalculated into idealized positions (riding model) and assigned temperature factors $H_{iso}(H)$ = 1.2 Ueq (pivot atom) or of 1.5 Ueq (methyl). H atoms in methyl, methylene, methine and hydrogen atoms in aromatic rings were placed with C-H distances of 0.99, 0.98, 0.97 and 0.95 Å, respectively, and 0.82 Å for OH.

Computational details

All calculations were carried out in the Gaussian16 quantum chemistry program.[S1] Geometry optimizations and analytical vibrational frequencies were carried out at the density functional level of theory using M06[S2] functional and implicit solvation model based on density (hexane, ε = 1.8819).[S3] Following double-ζ quality basis sets were used for all calculations: cc-pVDZ[S4] (for C, H, B, N, O), cc-pVDZ-PP including small-core relativistic pseudopotentials (for Ge) [S5] and LANL2DZ including small-core relativistic pseudopotentials (for K).[S6] Analytical vibrational frequencies within the harmonic approximation were computed to confirm a proper convergence to well-defined minima on the potential energy surface.

Figure S1. ^{1}H NMR spectrum of complex **2** in d_8-THF.

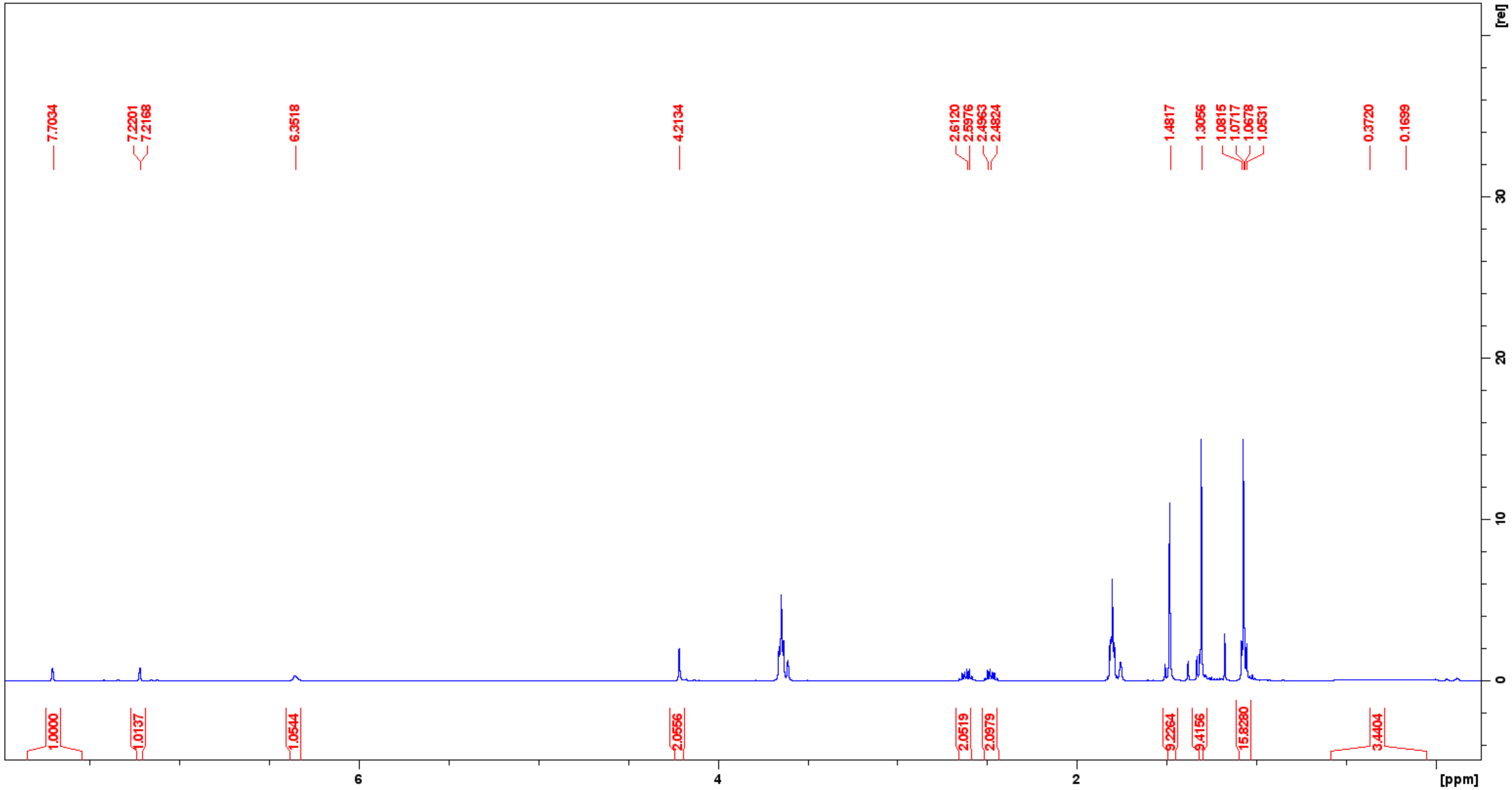

Figure S2. ^{13}C NMR spectrum of complex **2** in d_8-THF.

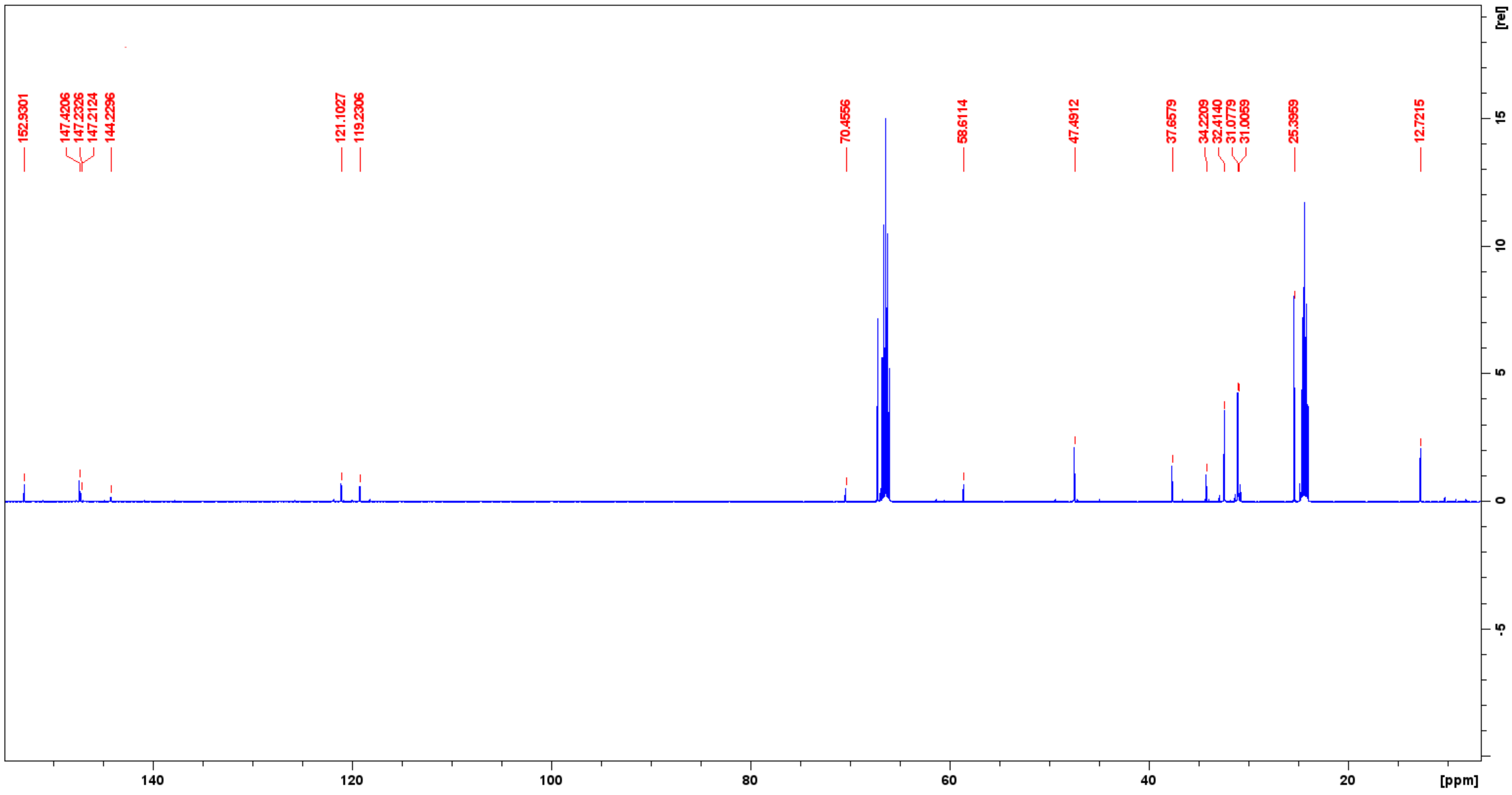

Figure S3. ^{11}B NMR spectrum of complex **2** in d_8-THF.

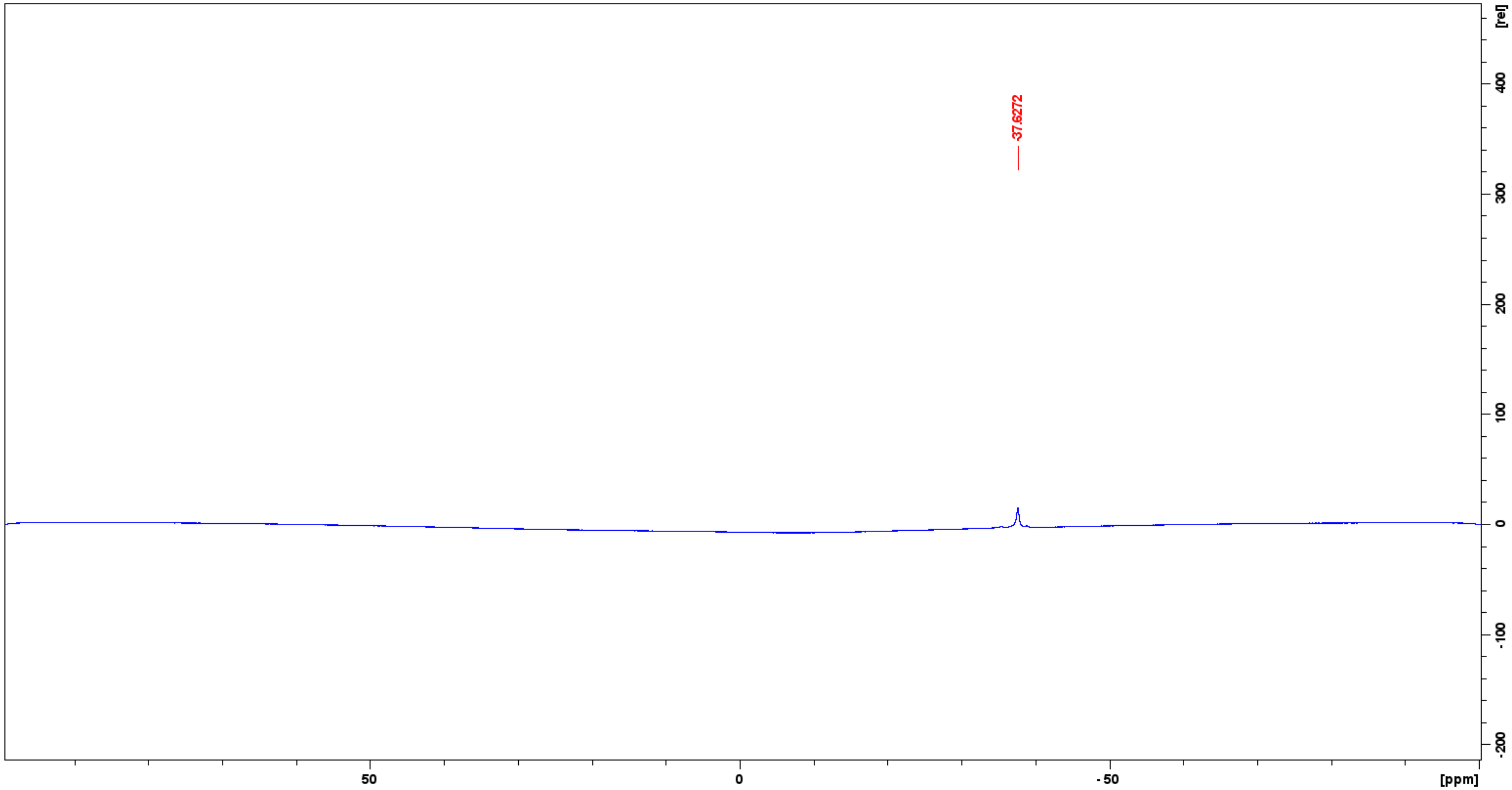

Figure S4. IR spectrum of complex **2**.

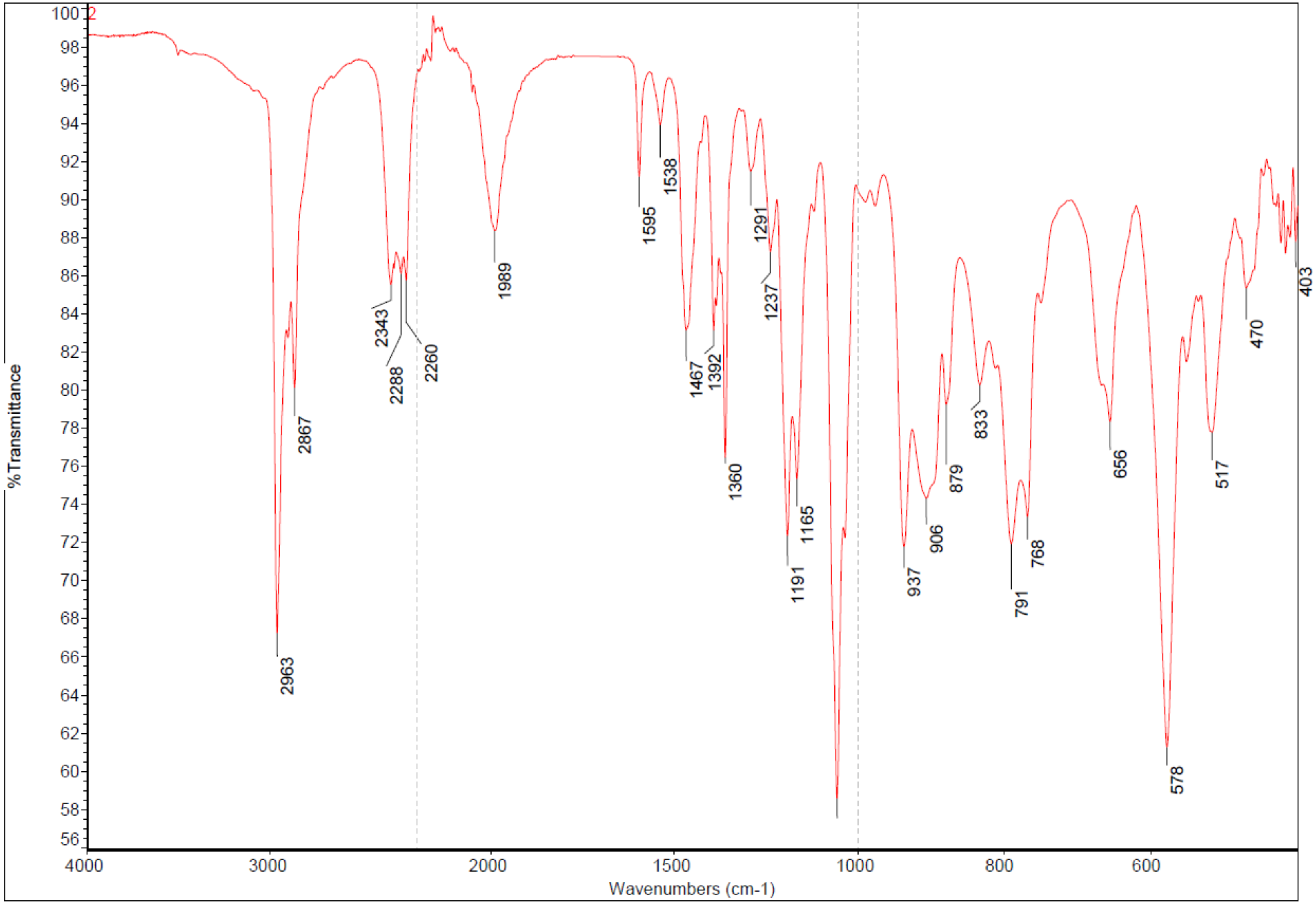

Figure S5. ^{1}H NMR spectrum of complex **3** in d_8-THF.

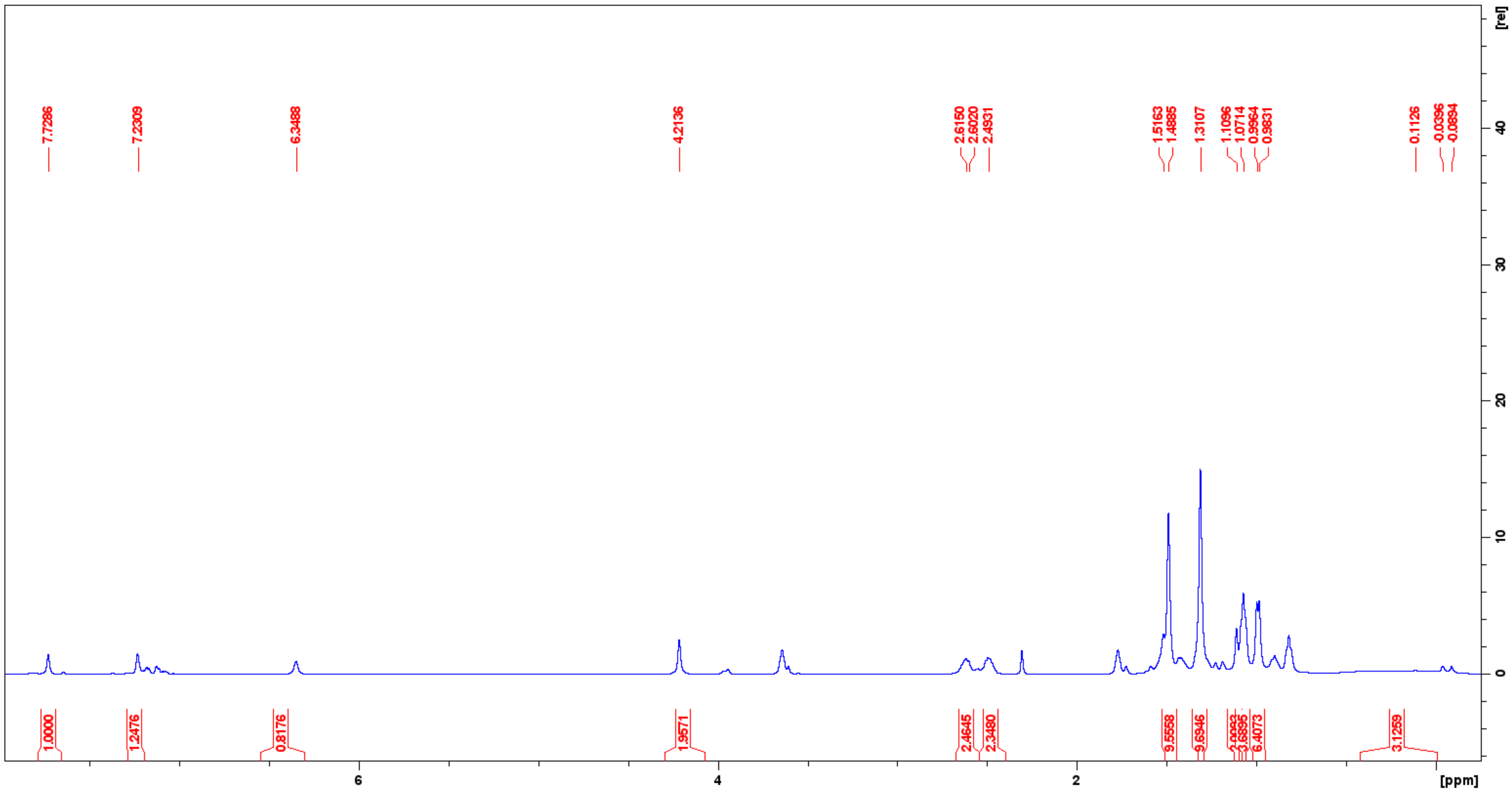

Figure S6. ^{13}C NMR spectrum of complex **3** in d_8-THF.

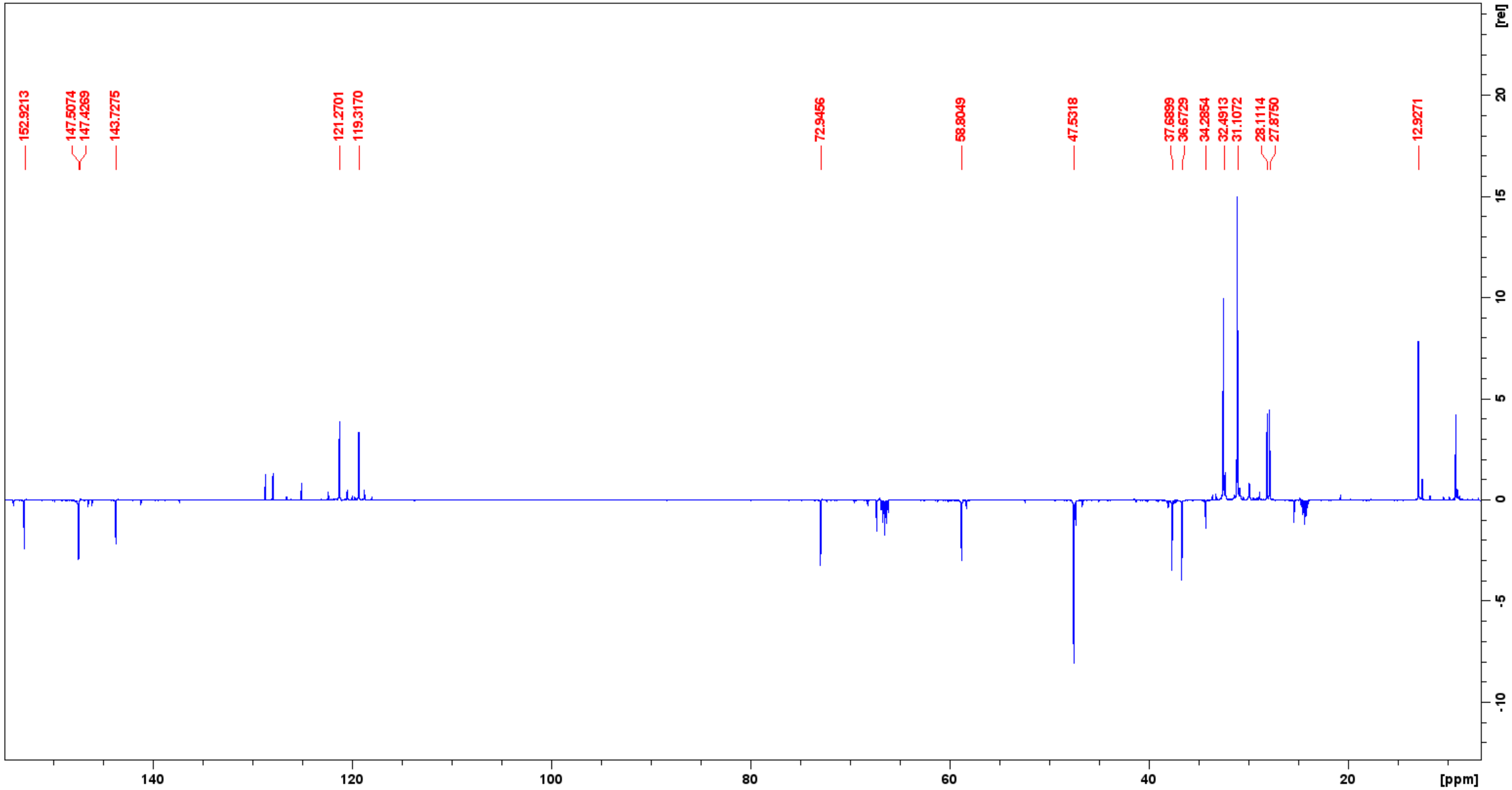

Figure S7. ^{11}B NMR spectrum of complex **3** in d_8-THF.

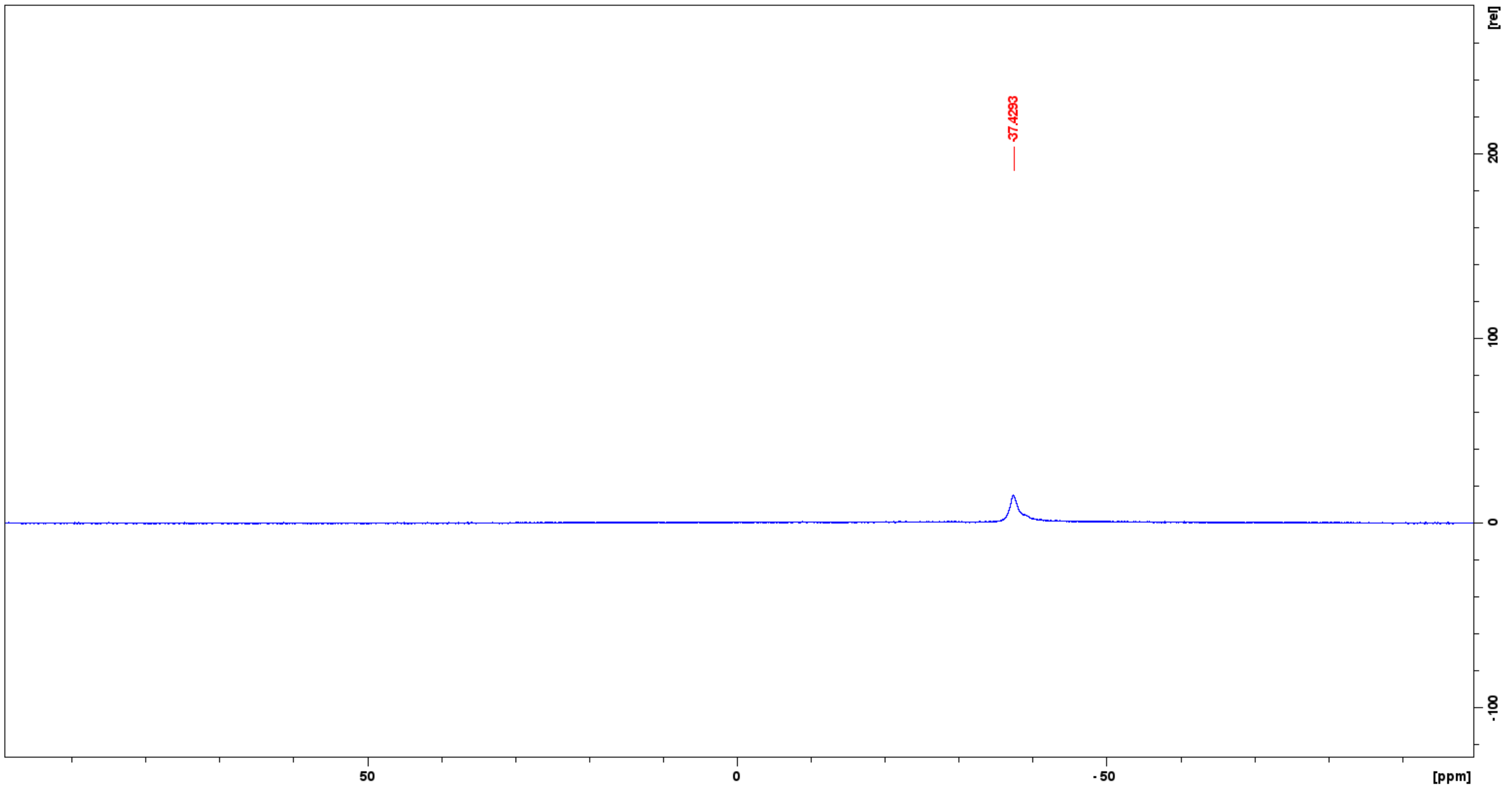

Figure S8. IR spectrum of complex **3**.

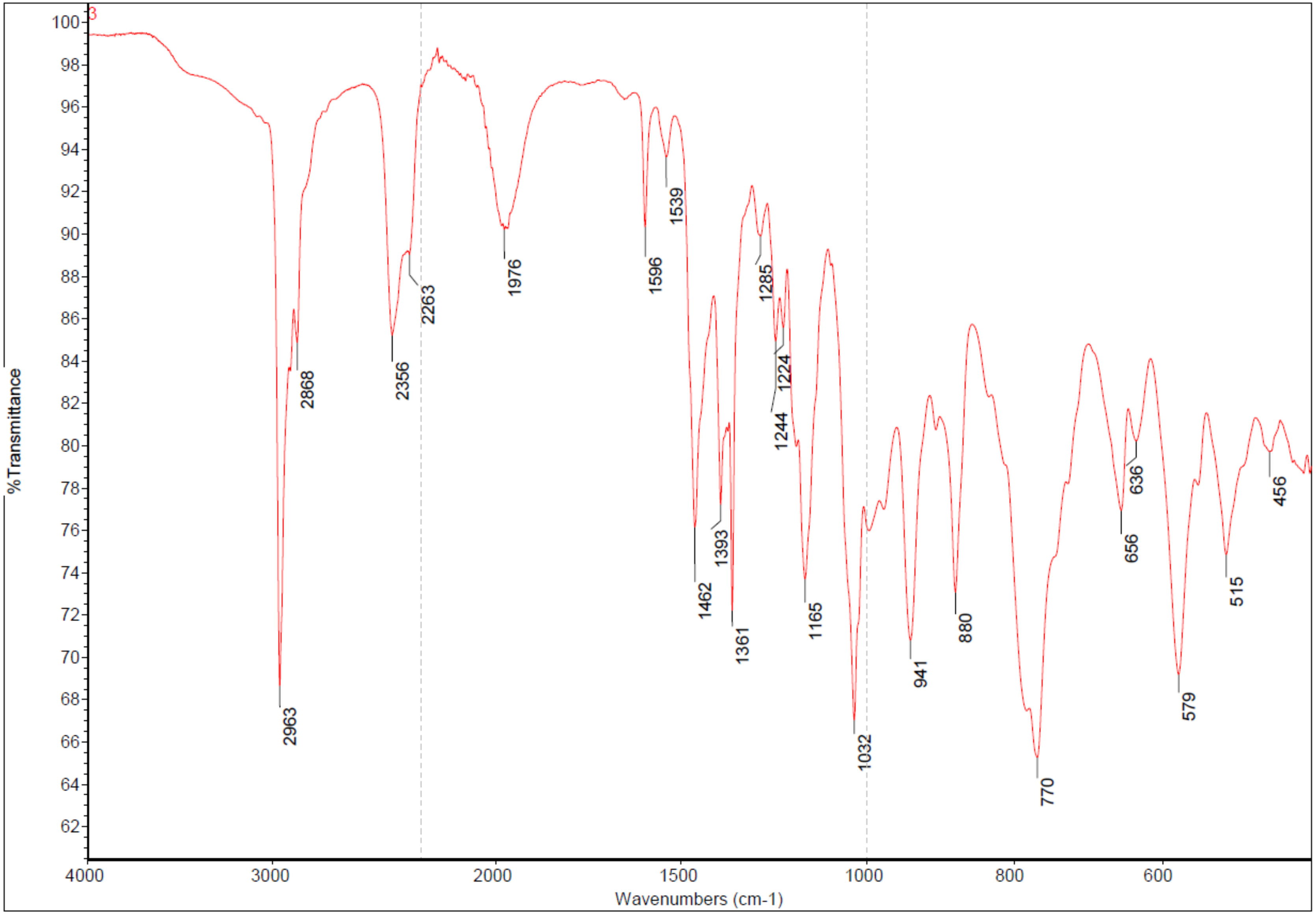

Figure S9. GC/MS analysis of a toluene solution of the stoichiometric reaction of **2** with benzaldehyde.

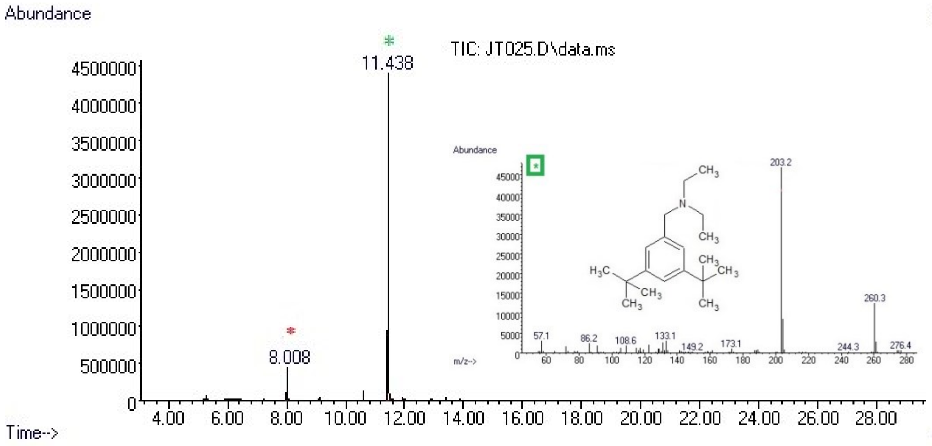

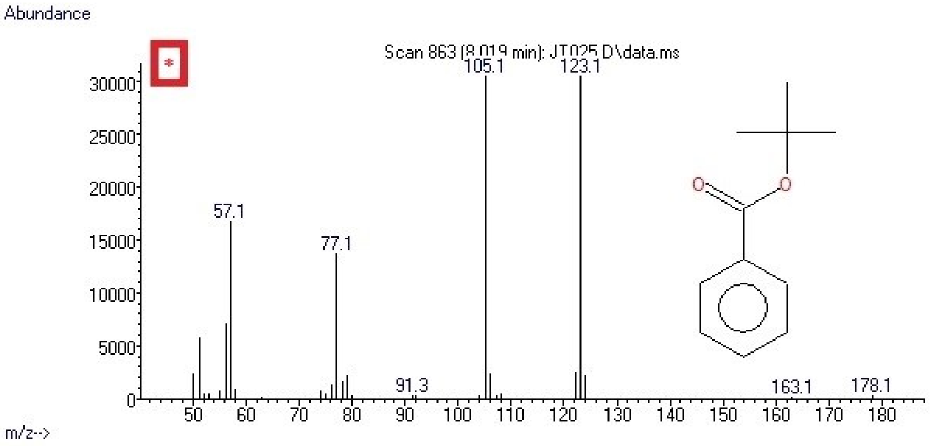

Figure S10. GC/MS analysis of a toluene solution of the stoichiometric reaction **3** with benzaldehyde.

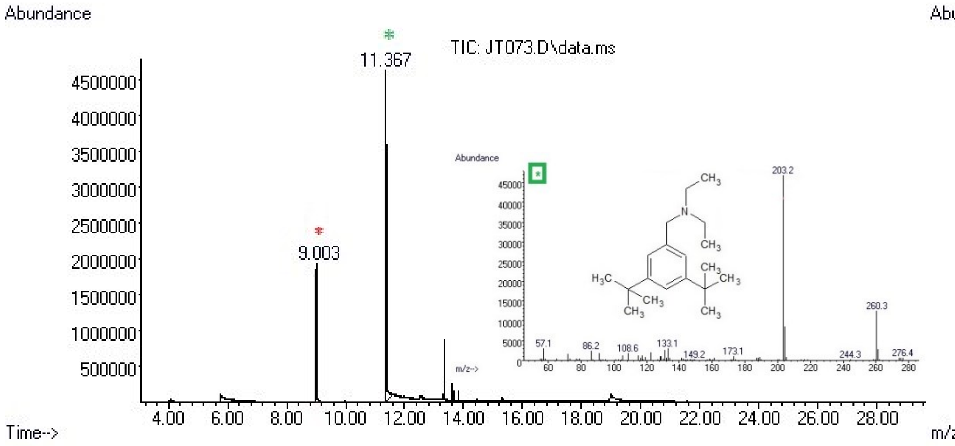

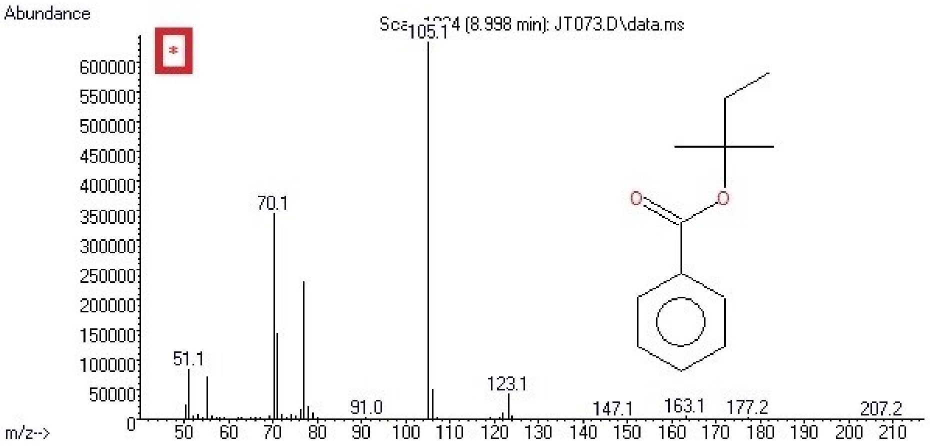

Figure S11. ^{1}H NMR spectrum of a reaction mixture of **2** with benzaldehyde in C_6D_6. We were able to observe signals matching shifts of **1**.

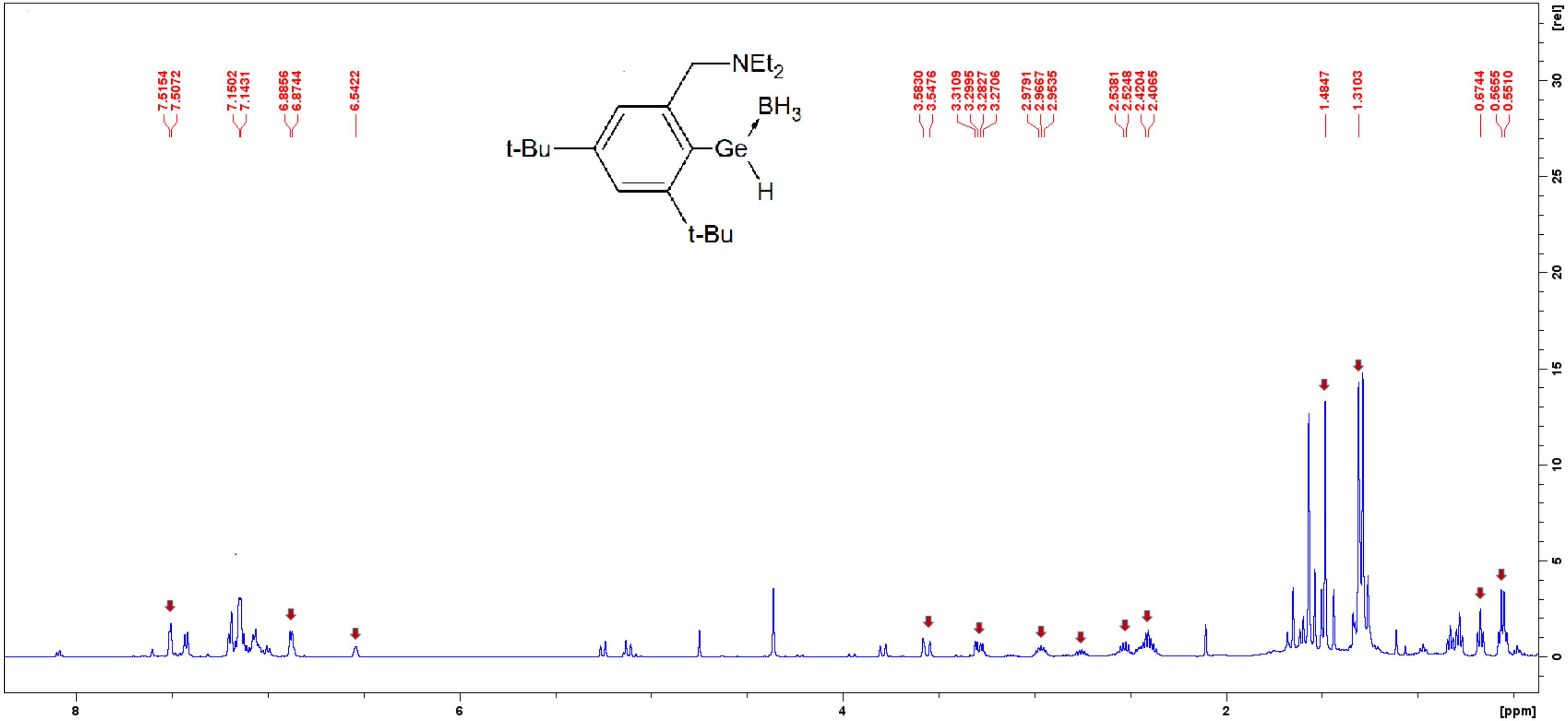

Figure S12. GC/MS analysis of a toluene solution of esterification of benzaldehyde and KO^{t}Bu in the presence of 10 mol. % of **1**.

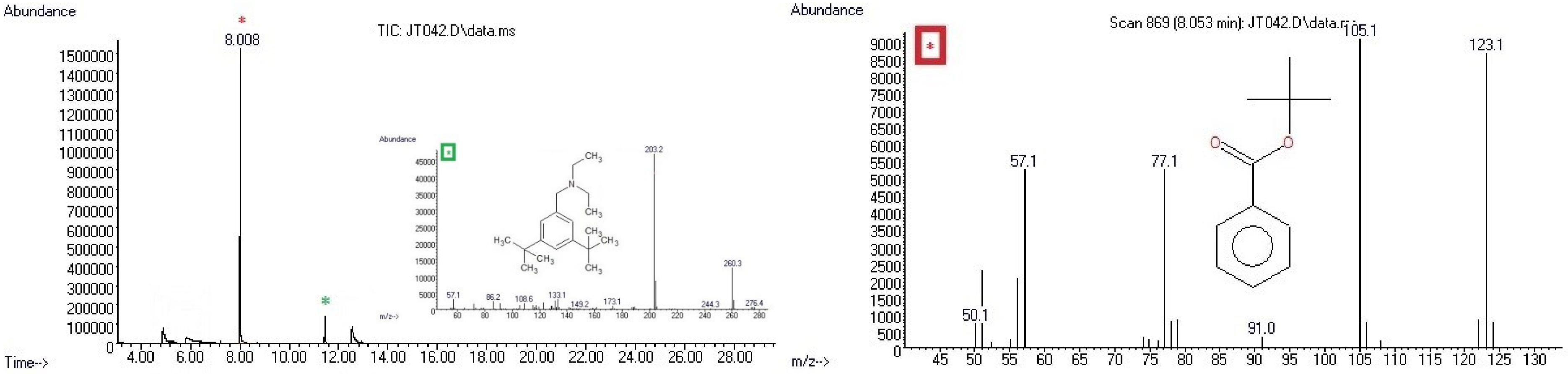

Figure S13. GC/MS analysis of a toluene solution of esterification of benzaldehyde and LiO^{t}Bu or NaO^{t}Bu in the presence of 10 mol. % of **1**.

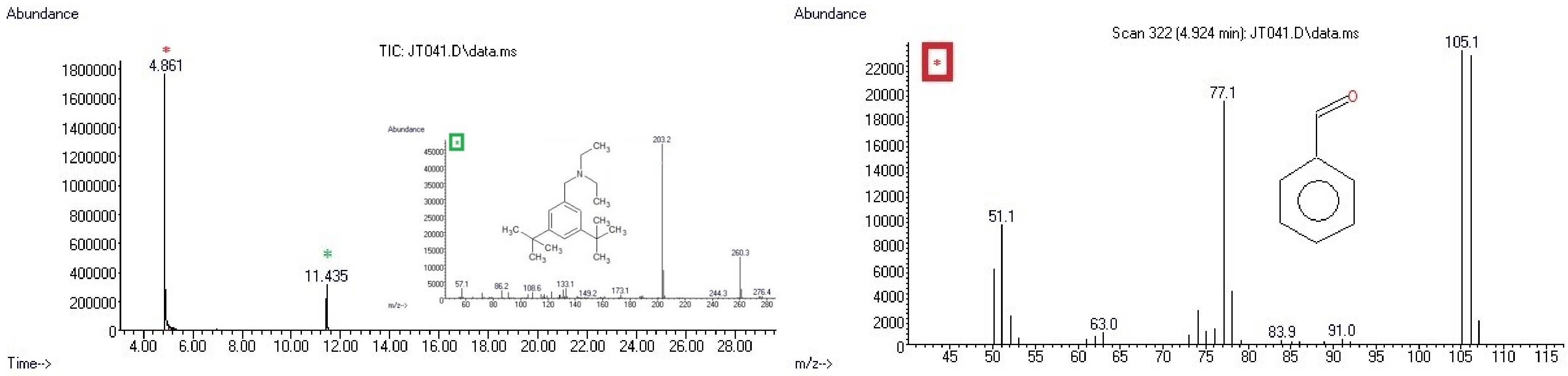

Figure S14. GC/MS analysis of a toluene solution of esterification of benzaldehyde and KO^{t}Bu in the presence of 1 mol. % of **1**.

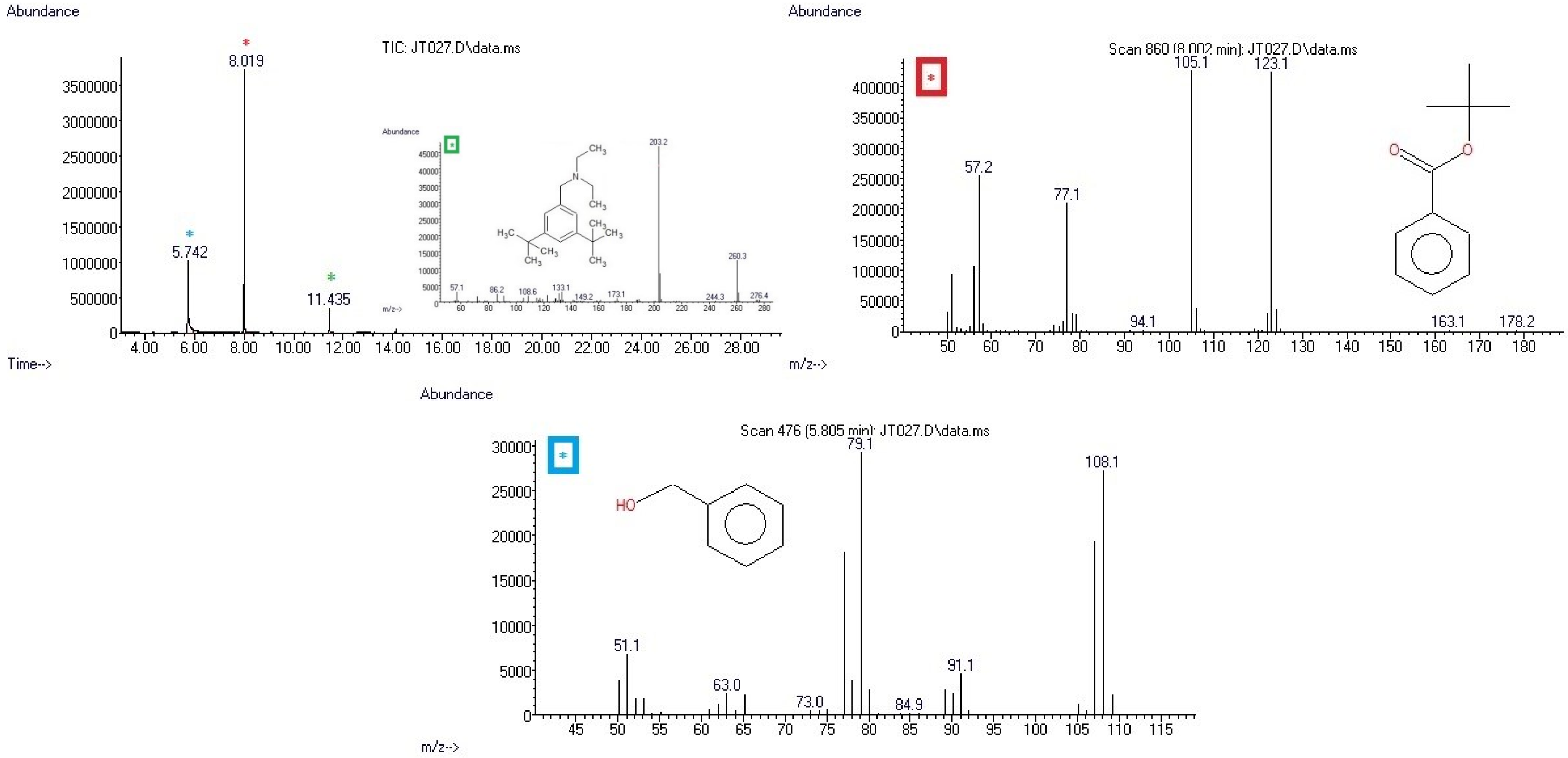

Figure S15. A) GC/MS analysis of a toluene solution of benzaldehyde and KO^{t}Bu without catalyst **1**. B) GC/MS analysis of a toluene solution of benzaldehyde and KO^{t}Bu with 10 mol % of BH_3 as catalyst

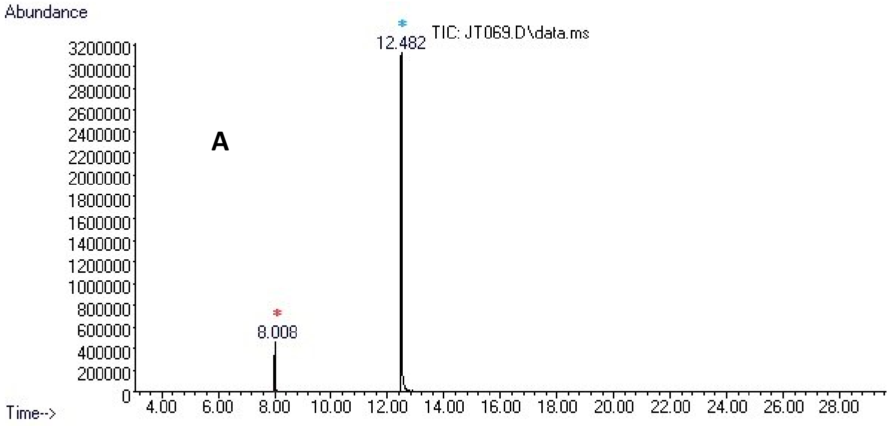

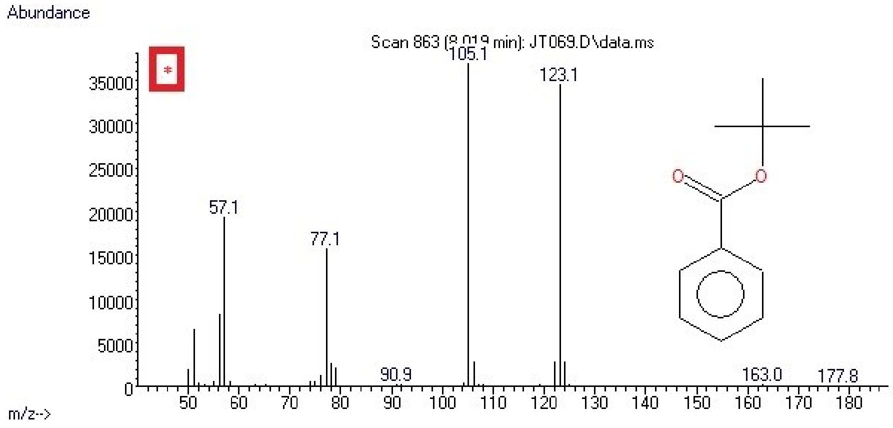

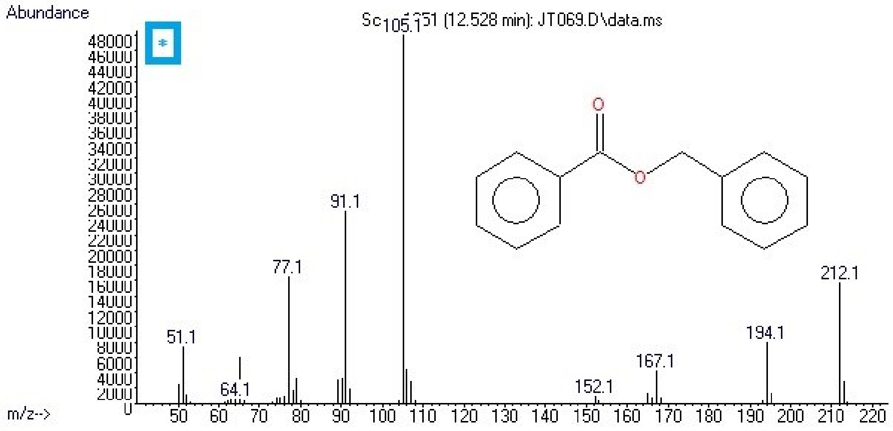

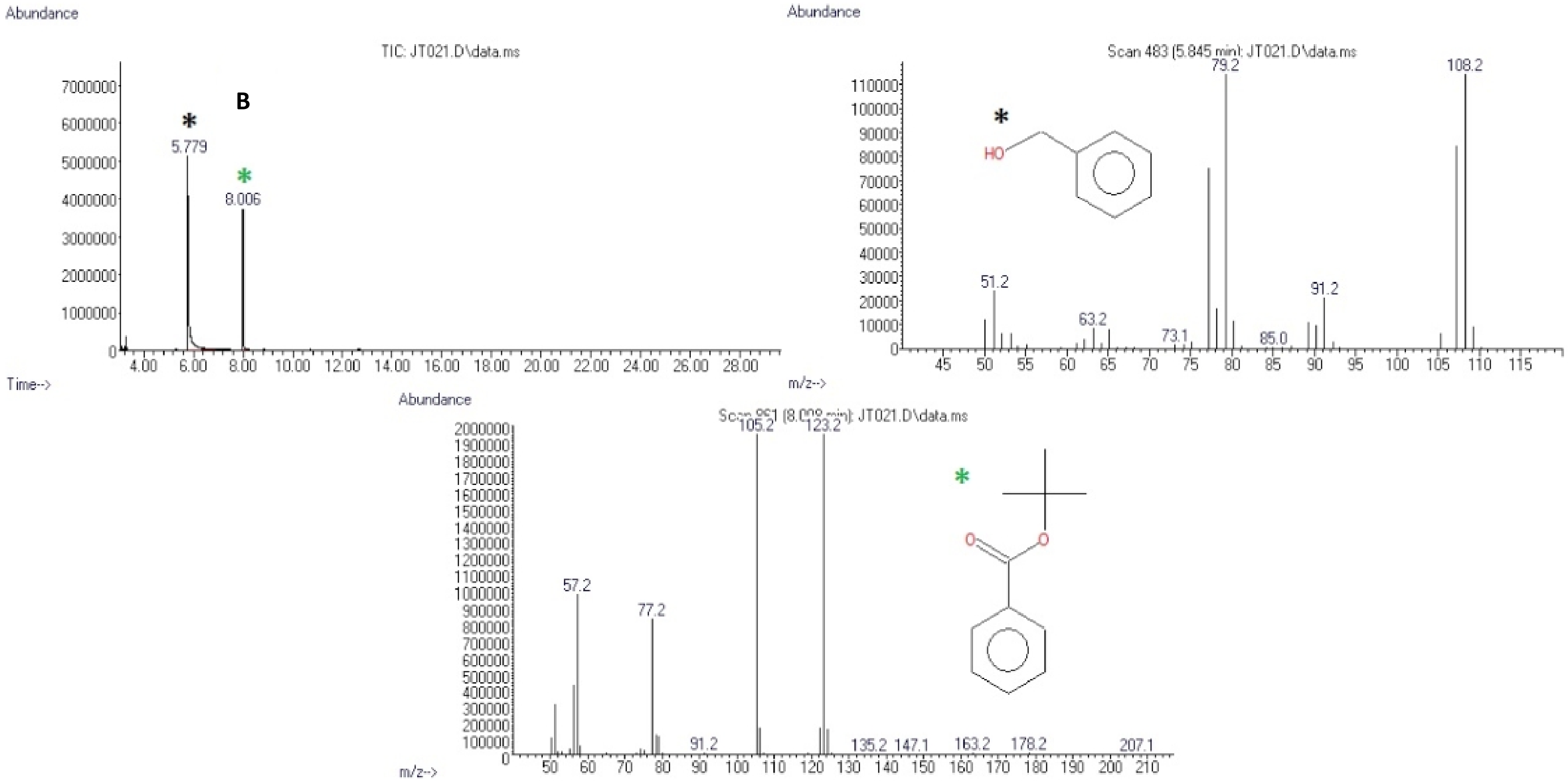
B
Abundance
TIC: JT021.D\data.ms
5.779
8.006
Time-->
Scan 483 (5.845 min): JT021.D\data.ms
79.2
108.2
51.2
63.2
73.1
85.0
91.2
HO
m/z-->
Scan 961 (8.008 min): JT021.D\data.ms
105.2
123.2
57.2
77.2
91.2
135.2
147.1
163.2
178.2
207.1
O
O

Figure S16. Time-resolved FT-IR study of benzaldehyde reaction with KO^{t}Bu and catalyst **1** at various molar ratio (benzaldehyde : KO^{t}Bu : catalyst).

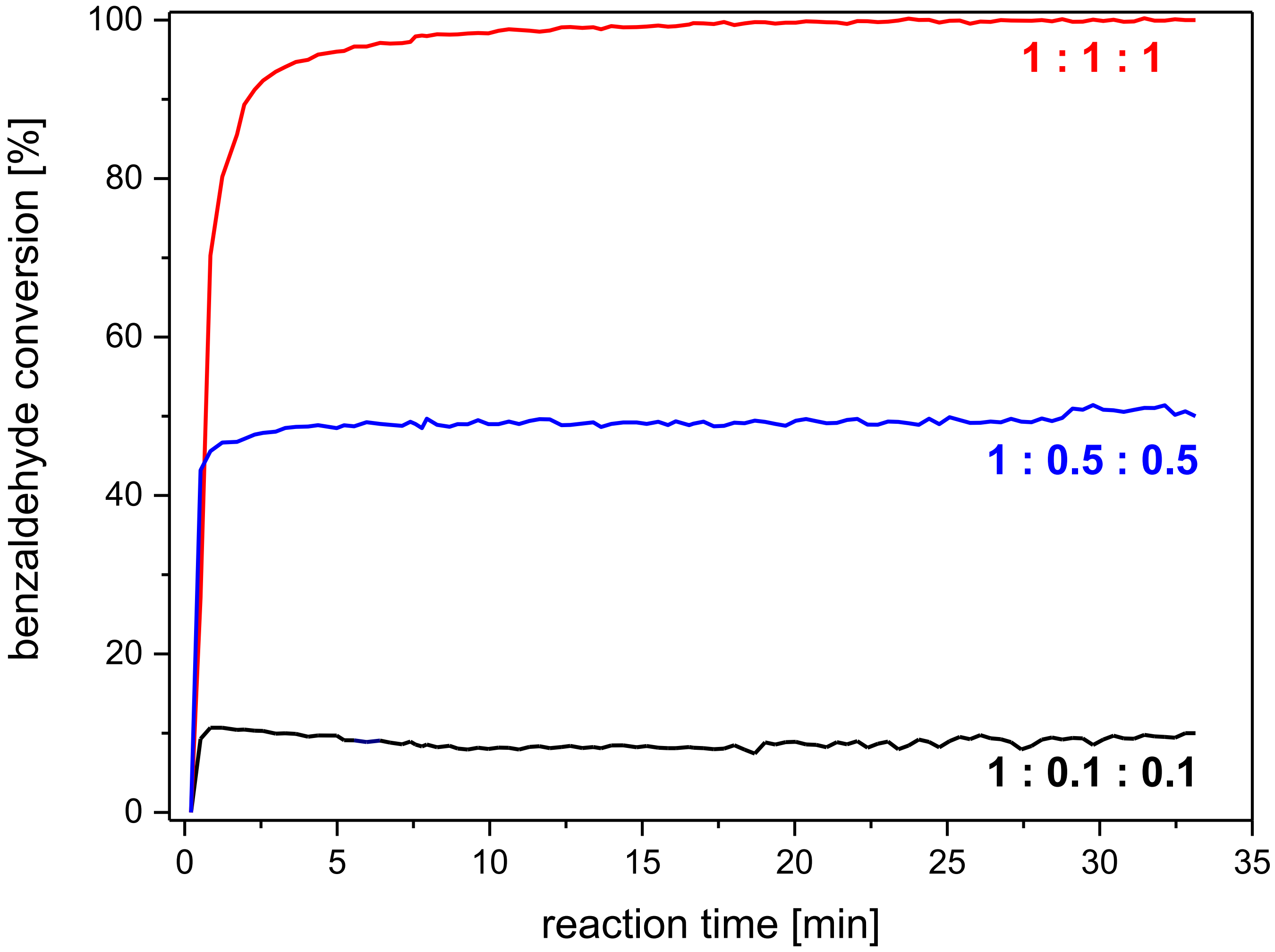

Figure S17. ^{11}B NMR spectrum of a stoichiometric reaction of **2** and benzaldehyde measured after 10 minutes at -50°C in d_8-THF.

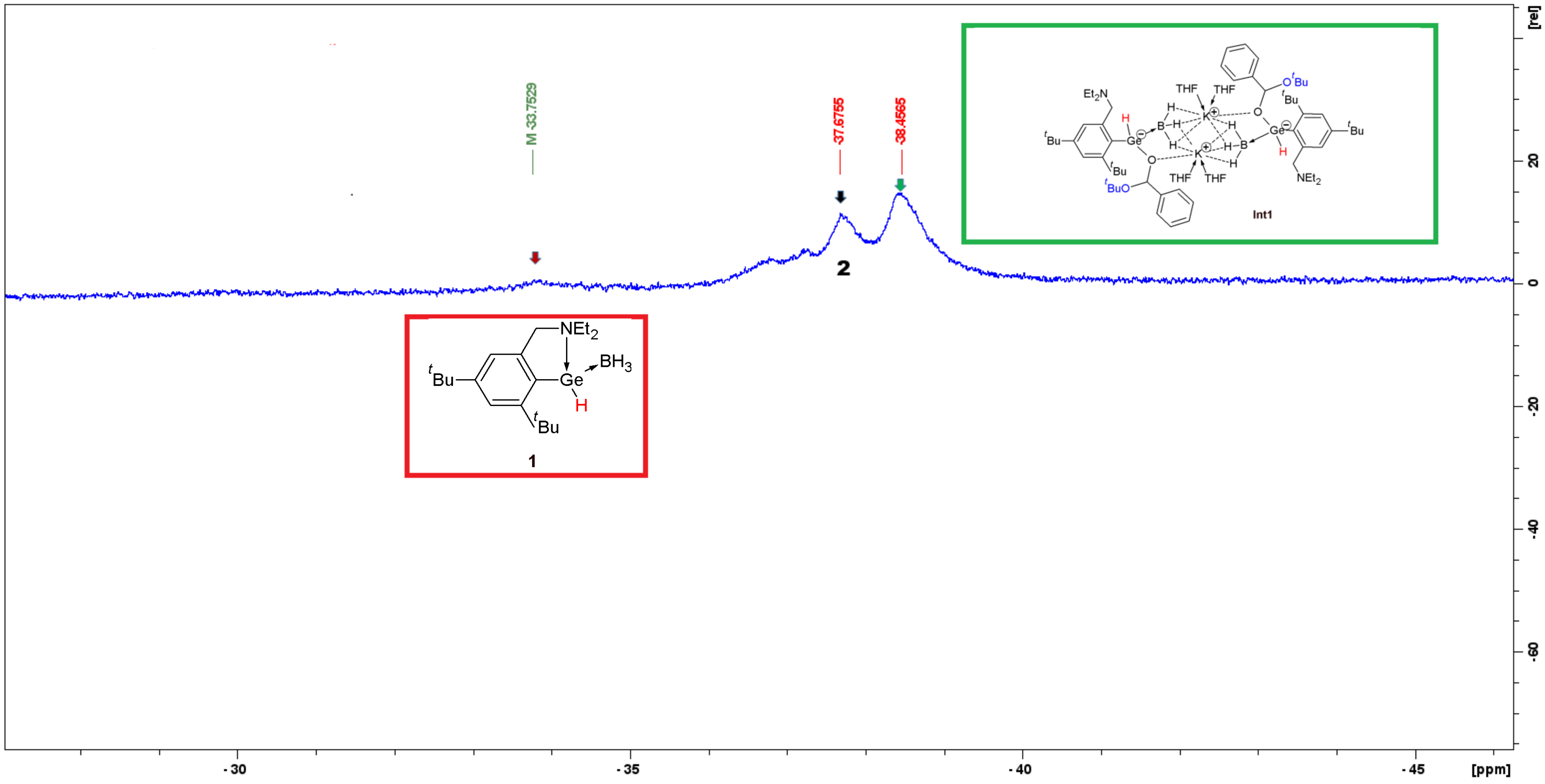

Figure S18. ^{11}B NMR spectrum of a stoichiometric reaction of **2** and benzaldehyde measured after 60 minutes at -50°C in d_8-THF.

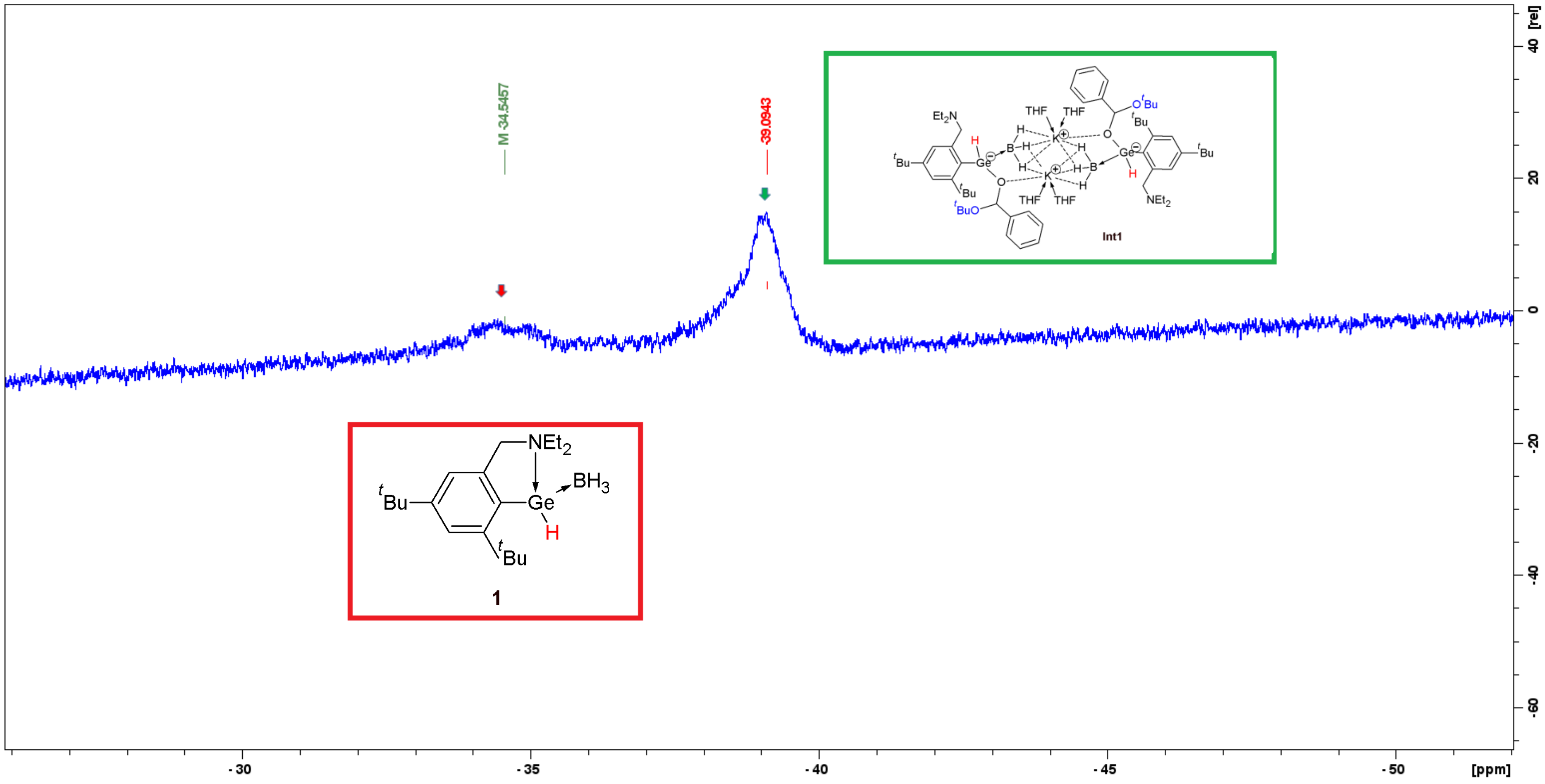

Figure S19. ^{11}B NMR spectrum of a mixture of esterification of benzaldehyde by KO^{t}Bu in the presence of 10 mol. % of **1** measured after 10 minutes at -50°C in d$_8$-THF.

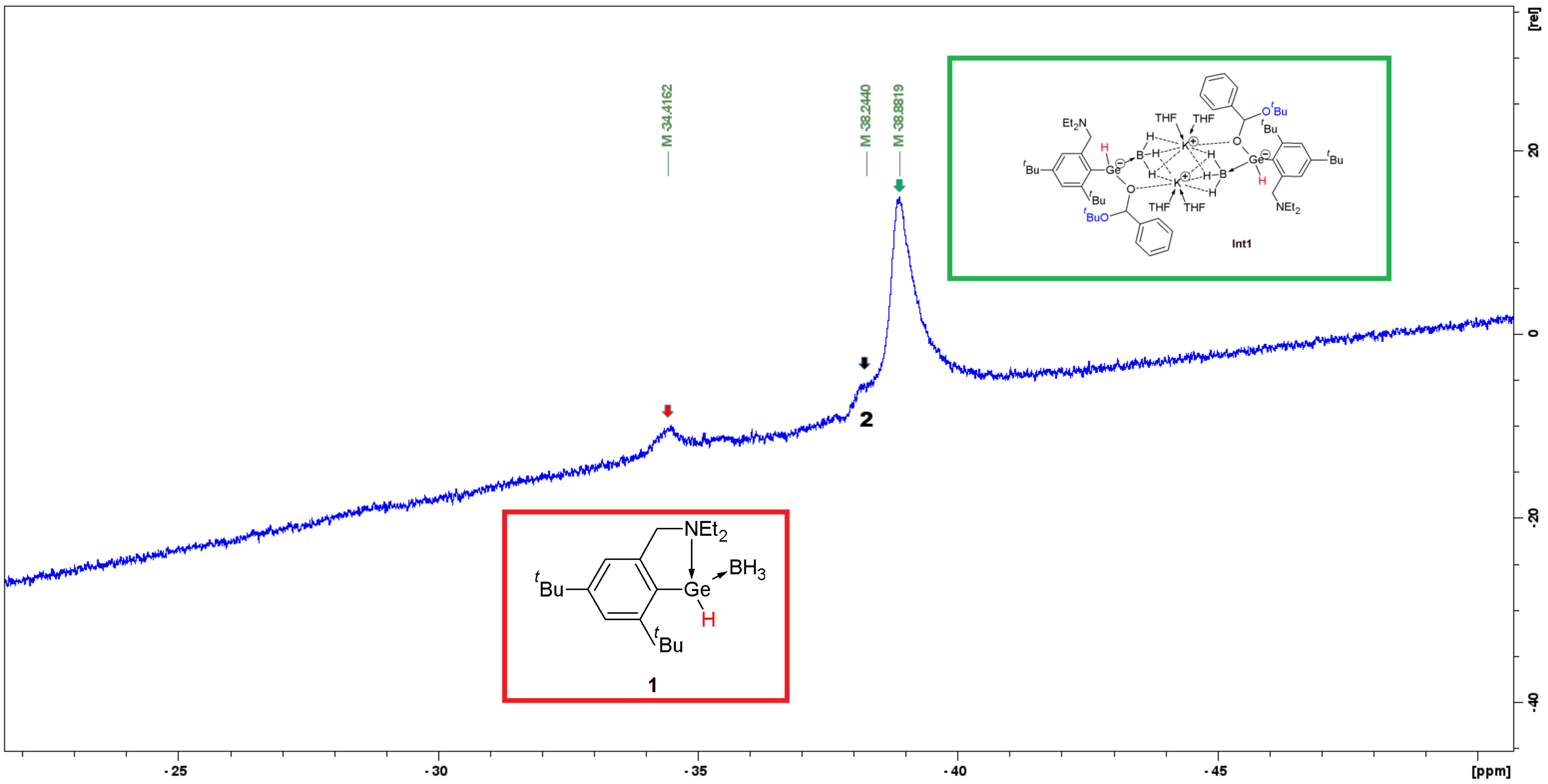

Figure S20. ^{11}B NMR spectrum of a mixture of esterification of benzaldehyde by KO^{t}Bu in the presence of 10 mol. % of **1** measured after 60 minutes at -50°C in d_8-THF.

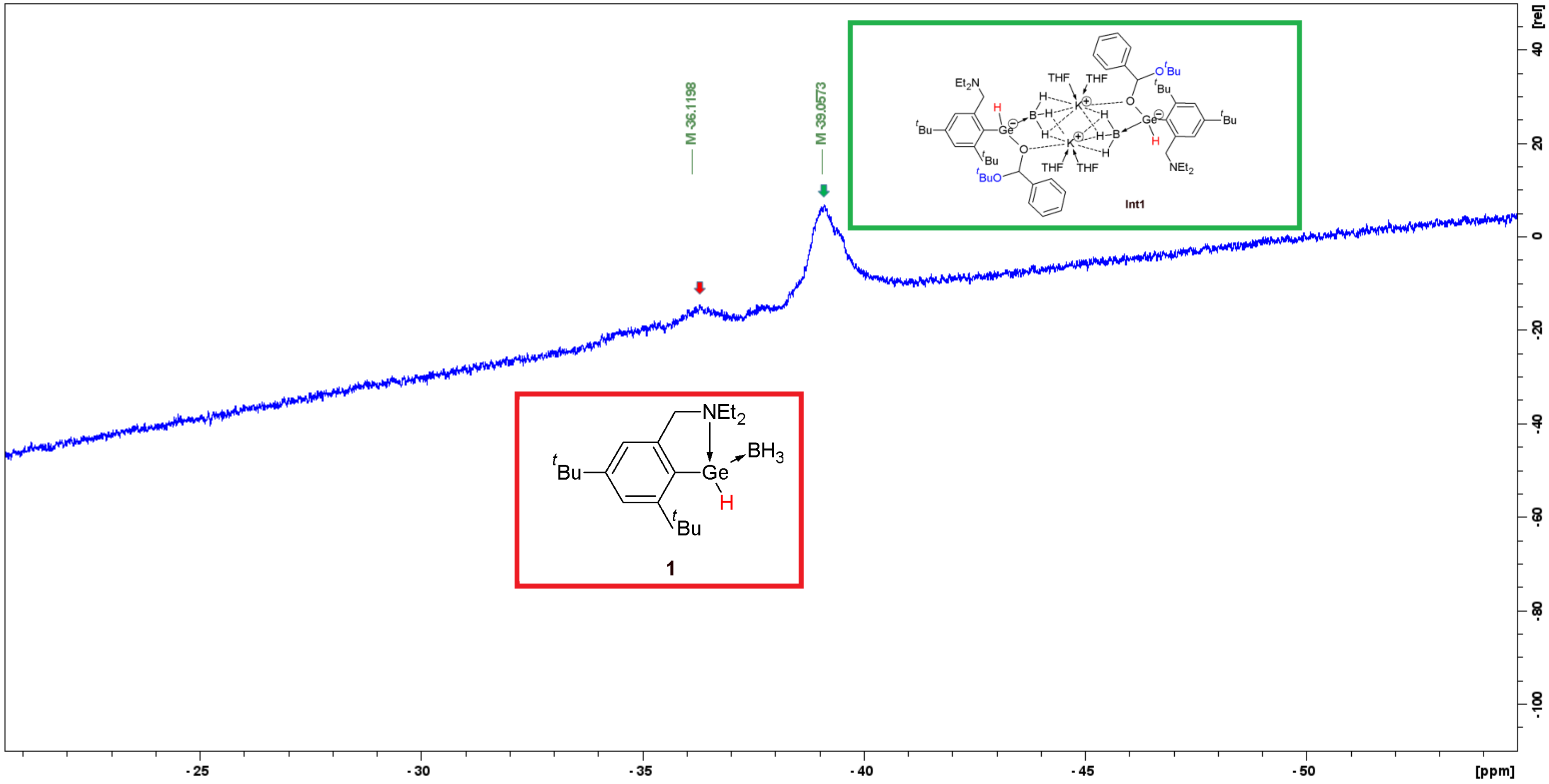

Figure S21. GC/MS analysis of a toluene solution of esterification of 4-nitrobenzaldehyde and KO^{t}Bu in the presence of 10 mol. % of **1**.

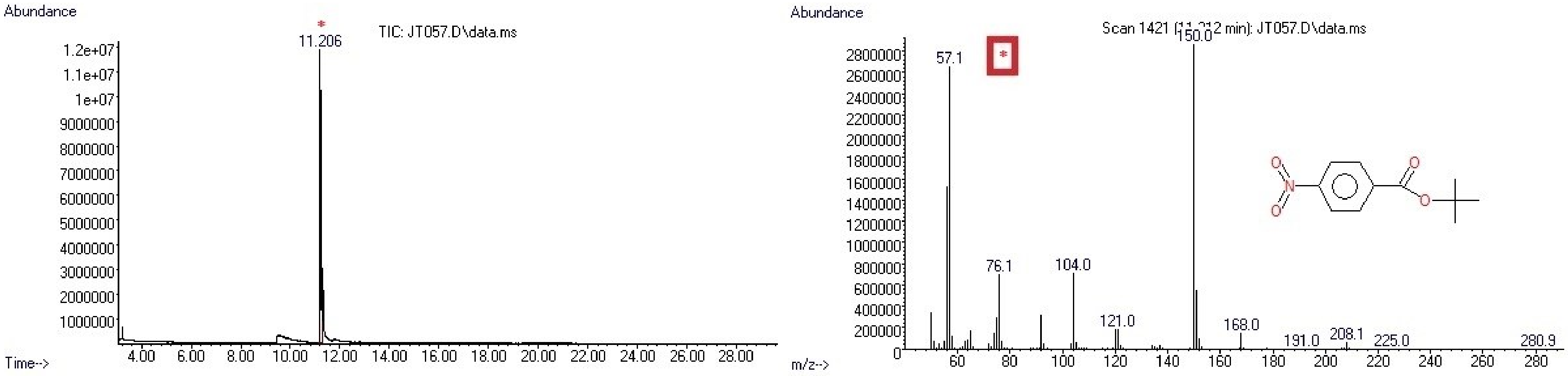

Figure S22. GC/MS analysis of a toluene solution of esterification of 4-cyanobenzaldehyde and KO^{t}Bu in the presence of 10 mol. % of **1**.

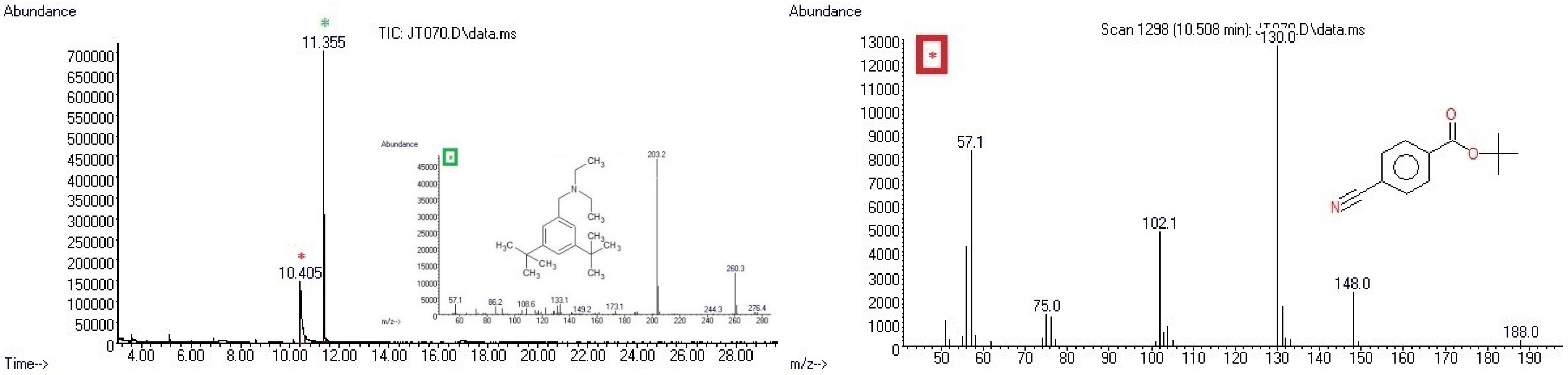

Figure S23. GC/MS analysis of toluene solution of esterification of 4-methoxybenzaldehyde and KO^{t}Bu in the presence of 10 mol. % of **1**.

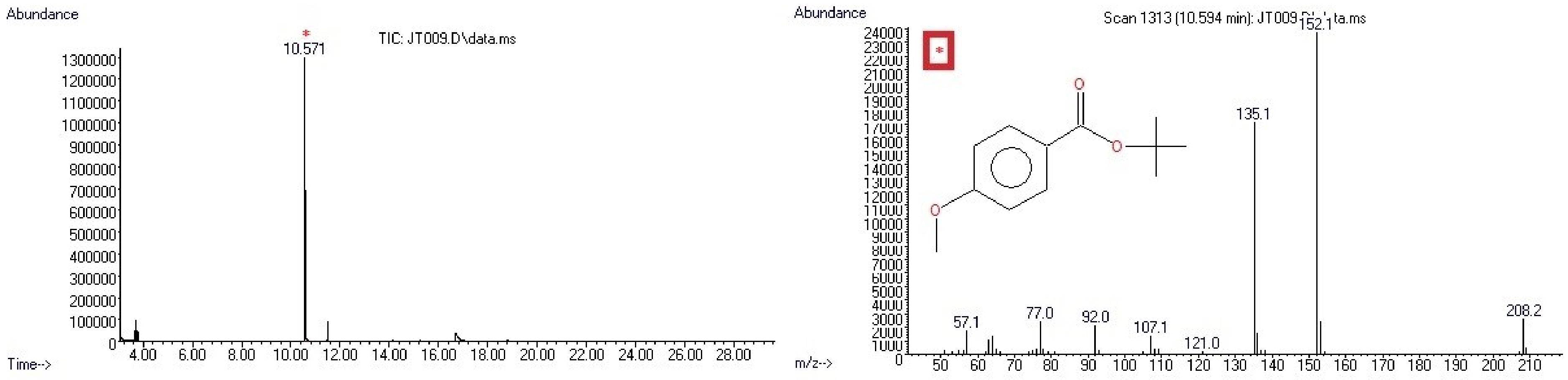

Figure S24. GC/MS analysis of a toluene solution of esterification of 2-thiophenecarboxaldehyde and KO^{t}Bu in the presence of 10 mol. % of **1**.

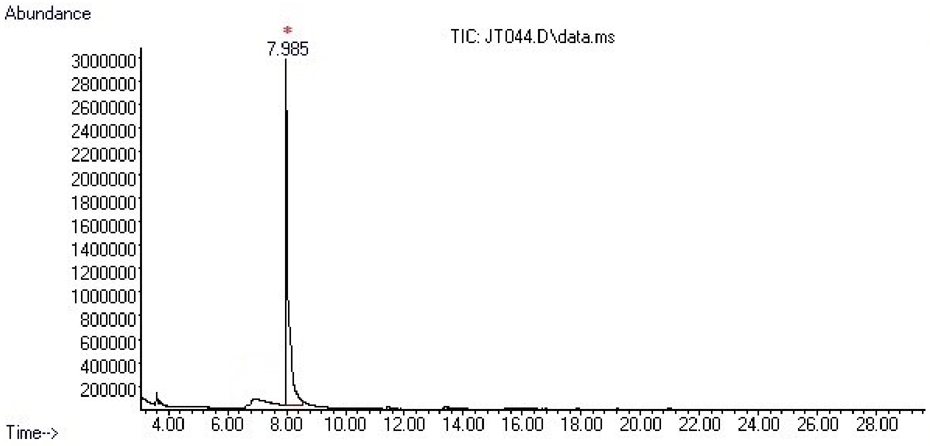

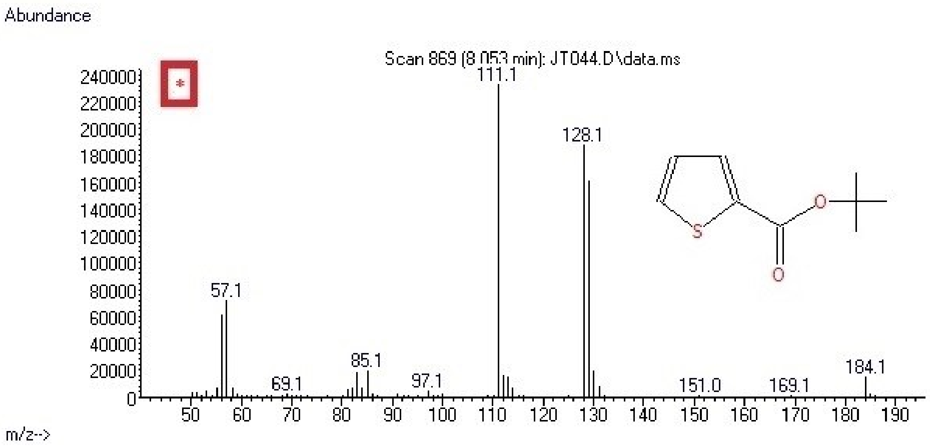

Figure S25. ^{1}H NMR spectrum of *tert*-butoxy ester of 4-cyanobenzoic acid in C_6D_6.

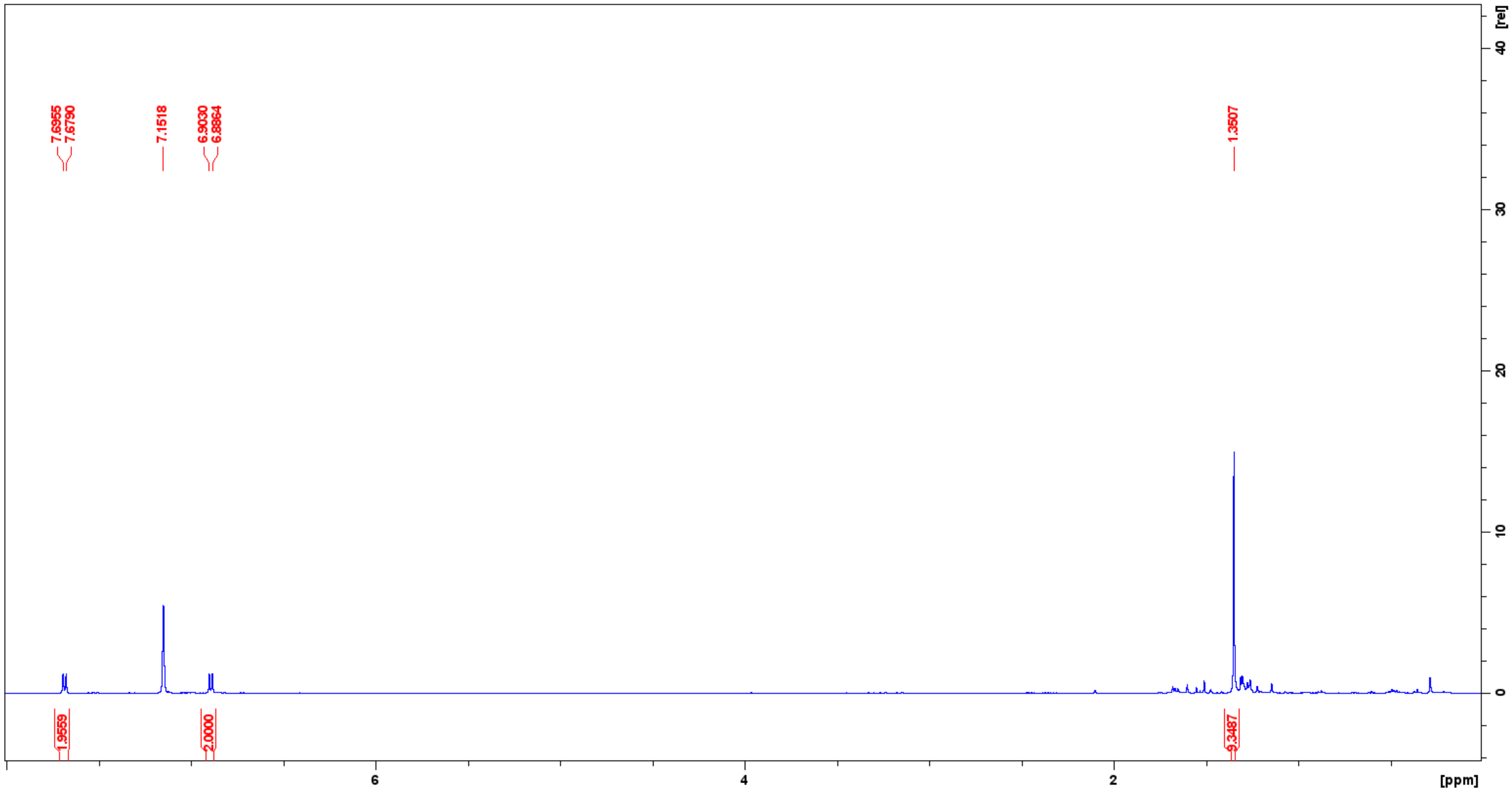

Figure S26. Figure of Schlenk flask with heterogenous mixture after the catalytical experiment. The solid was filtrated and characterized as KH.

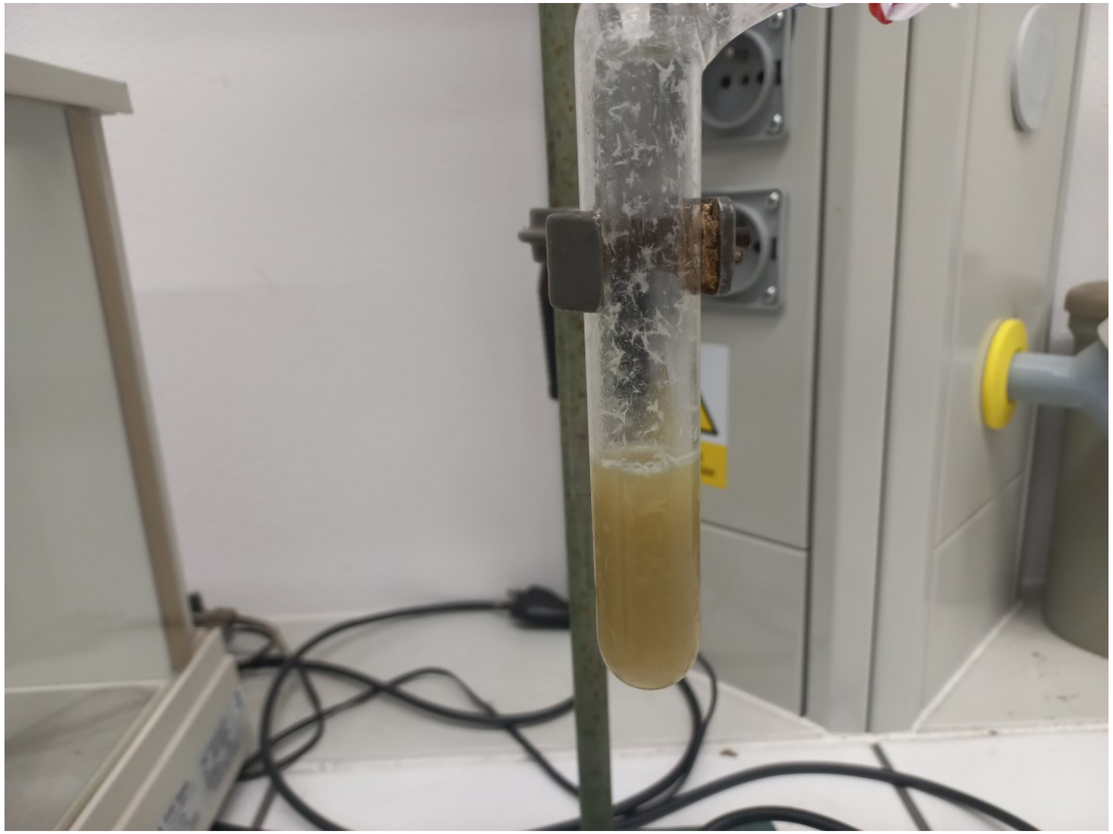

Figure S27. Raman spectra of mineral oil (**top**), commercial KH dispersion in mineral oil (**mid**) and precipitate isolated from catalyzed reaction of benzaldehyde with KOtBu (**bottom**). Asterisks denotes bands characteristic for potassium hydride.

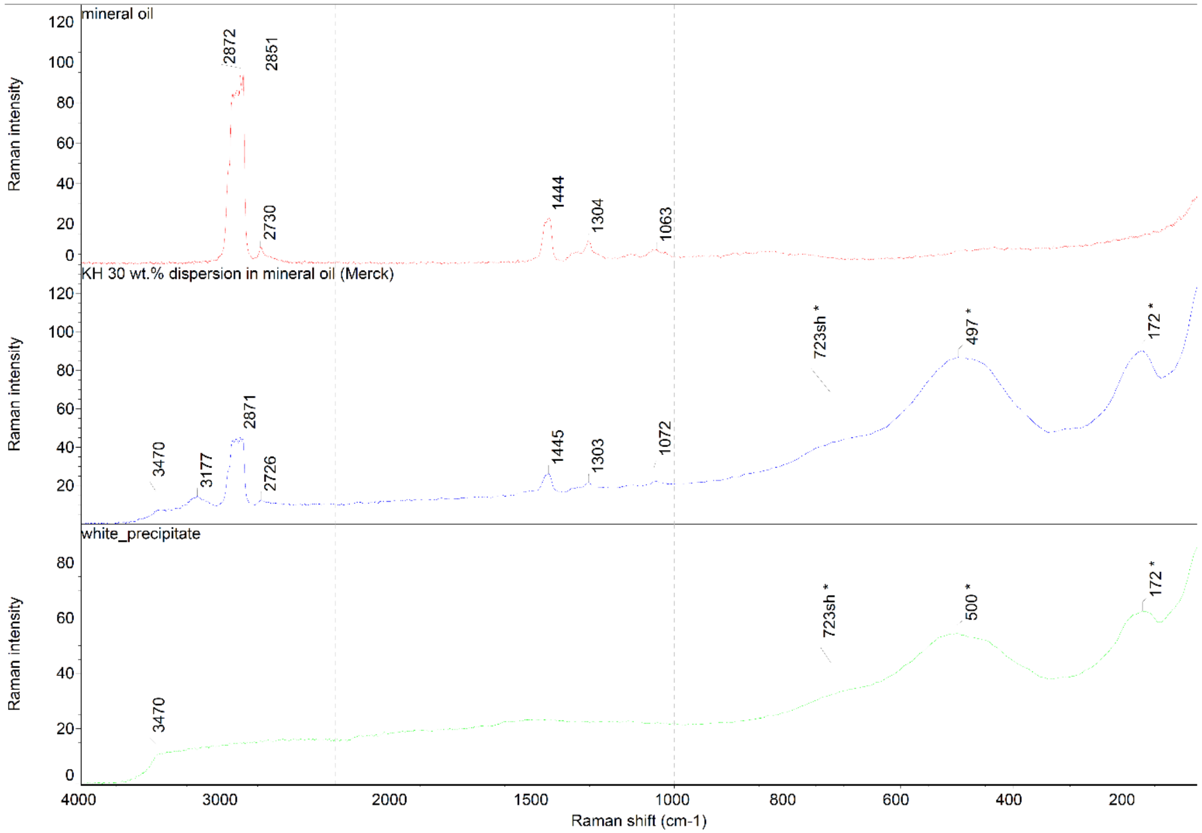

Table S1: Crystalographic parameters of **2** and **3**

	2	
a	17.8963(5)	18.0564(10)
b	20.9390(7)	20.9749(11)
c	22.6283(6)	22.6797(10)
Alpha	90	90
Beta	90	90
Gamma	90	90
Volume	8479.5(4)	8589.5(8)
Space group	P b c a	P b c a
Moiety formula	C62 H122 B2 Ge2 K2 N2 O6, 3(C4 H8 O)	C64 H126 B2 Ge2 K2 N2 O6,3(C4 H8 O)
Sum formula	C74 H146 B2 Ge2 K2 N2 O9	C76 H150 B2 Ge2 K2 N2 O9
Z	4	4
F000’	2995.70	3059.72
Temperature	150 K	150 K
Hall group	-P 2ac 2ab	-P 2ac 2ab
Tmin,Tmax	0.666,0.746	0.384,0.746
R(reflections)	0.0462(6424)	0.0610(6727)
Mr	1452.92	1480.97
μ(mm-1)	0.856	0.846
h,k,lmax	22,26,28	22,26,28
Tmin’	0.595	0.384
Dx,g cm-3	1.138	1.145
Theta(max)	26.499	26.500
S	1.076	0.933
Data completeness	0.997	0.995
wR2(reflections)	0.1329(8768)	0.1757(8866)

Benzaldehyde

Standard orientation:

Center Number	Atomic Number	Coordinates (Angstroms) X	Y	Z
1	6	0.000031404	0.000000000	0.000796236
2	6	-0.000108020	0.000000000	-0.000182962
3	6	0.000529981	0.000000000	0.000143224
4	6	-0.000155714	0.000000000	0.000099048
5	6	-0.000395183	0.000000000	0.000036299
6	6	0.000244969	0.000000000	-0.000072013
7	1	-0.000101781	0.000000000	-0.000180428
8	1	-0.000167014	0.000000000	-0.000281034
9	1	0.000037647	0.000000000	0.000049212
10	1	-0.000068057	0.000000000	0.000051333
11	1	-0.000043259	0.000000000	0.000079624
12	6	0.002233340	0.000000000	-0.002440433
13	1	-0.000623448	0.000000000	-0.000003220
14	8	-0.001414867	0.000000000	0.001905114

Sum of electronic and thermal Free Energies = -345.2604570 A.U./particle

Complex 2

Standard orientation:

Center Number	Atomic Number	Atomic Type	Coordinates (Angstroms) X	Y	Z
1	19	0	-1.589047	-0.867933	-0.981876
2	5	0	1.258922	-0.418625	-1.877024
3	1	0	0.804844	-0.813398	-2.951068
4	1	0	1.119848	-1.276626	-0.987651
5	1	0	0.761505	0.659968	-1.557814
6	19	0	1.589047	0.867933	0.981876
7	5	0	-1.258922	0.418625	1.877024
8	1	0	-0.804844	0.813398	2.951068
9	1	0	-0.761505	-0.659968	1.557814
10	1	0	-1.119848	1.276626	0.987651
11	32	0	-3.310541	0.237130	2.025458
12	1	0	-4.080169	1.592760	1.854940
13	32	0	3.310541	-0.237130	-2.025458
14	1	0	4.080169	-1.592760	-1.854940
15	8	0	-3.860310	-0.567541	0.380147
16	8	0	3.860310	0.567541	-0.380147
17	6	0	5.182649	0.647860	-0.006401
18	6	0	-5.182649	-0.647860	0.006401
19	8	0	1.253933	3.327839	2.110818
20	8	0	-1.253933	-3.327839	-2.110818
21	6	0	-4.489310	-0.682463	3.377589
22	6	0	-6.671790	-1.928043	4.745930
23	6	0	-4.809252	-2.043886	3.177327
24	6	0	-5.179932	0.006052	4.411562
25	6	0	-6.268210	-0.622511	5.028001
26	6	0	-5.878415	-2.641788	3.854369
27	1	0	-6.835820	-0.065977	5.777097
28	1	0	-6.081798	-3.698570	3.655475
29	6	0	4.489310	0.682463	-3.377589
30	6	0	6.671790	1.928043	-4.745930
31	6	0	4.809252	2.043886	-3.177327
32	6	0	5.179932	-0.006052	-4.411562
33	6	0	6.268210	0.622511	-5.028001
34	6	0	5.878415	2.641788	-3.854369
35	1	0	6.835820	0.065977	-5.777097
36	1	0	6.081798	3.698570	-3.655475
37	6	0	-7.901407	-2.514693	5.434318
38	6	0	7.901407	2.514693	-5.434318
39	6	0	7.699608	2.508558	-6.952838
40	1	0	8.582796	2.935103	-7.459580
41	1	0	6.819817	3.111895	-7.233748
42	1	0	7.548337	1.490749	-7.347103
43	6	0	8.176028	3.950903	-4.997866
44	1	0	8.349300	4.023402	-3.911109
45	1	0	7.344950	4.626907	-5.259342
46	1	0	9.080134	4.327551	-5.504432
47	6	0	9.126265	1.666169	-5.077562
48	1	0	9.297043	1.663493	-3.987396
49	1	0	10.030692	2.070493	-5.564729

50	1	0	9.011213	0.618974	-5.401108
51	6	0	-7.699608	-2.508558	6.952838
52	1	0	-7.548337	-1.490749	7.347103
53	1	0	-6.819817	-3.111895	7.233748
54	1	0	-8.582796	-2.935103	7.459580
55	6	0	-9.126265	-1.666169	5.077562
56	1	0	-9.011213	-0.618974	5.401108
57	1	0	-10.030692	-2.070493	5.564729
58	1	0	-9.297043	-1.663493	3.987396
59	6	0	-8.176028	-3.950903	4.997866
60	1	0	-7.344950	-4.626907	5.259342
61	1	0	-8.349300	-4.023402	3.911109
62	1	0	-9.080134	-4.327551	5.504432
63	6	0	-4.811048	1.410120	4.939888
64	6	0	4.811048	-1.410120	-4.939888
65	6	0	-3.322424	1.724965	4.771784
66	1	0	-2.689526	0.905488	5.154204
67	1	0	-3.071225	2.639011	5.336661
68	1	0	-3.031032	1.928095	3.731702
69	6	0	-5.655991	2.479262	4.239445
70	1	0	-5.471697	2.492529	3.153470
71	1	0	-5.418026	3.481188	4.639313
72	1	0	-6.732393	2.296384	4.402606
73	6	0	-5.082935	1.511535	6.449210
74	1	0	-4.582266	0.700153	7.004378
75	1	0	-6.153808	1.489228	6.702646
76	1	0	-4.693387	2.471867	6.826085
77	6	0	5.082935	-1.511535	-6.449210
78	1	0	6.153808	-1.489228	-6.702646
79	1	0	4.582266	-0.700153	-7.004378
80	1	0	4.693387	-2.471867	-6.826085
81	6	0	3.322424	-1.724965	-4.771784
82	1	0	3.071225	-2.639011	-5.336661
83	1	0	2.689526	-0.905488	-5.154204
84	1	0	3.031032	-1.928095	-3.731702
85	6	0	5.655991	-2.479262	-4.239445
86	1	0	6.732393	-2.296384	-4.402606
87	1	0	5.418026	-3.481188	-4.639313
88	1	0	5.471697	-2.492529	-3.153470
89	6	0	4.047739	2.946952	-2.235755
90	1	0	4.366843	3.998676	-2.431167
91	1	0	4.338522	2.741594	-1.190777
92	6	0	-4.047739	-2.946952	2.235755
93	1	0	-4.338522	-2.741594	1.190777
94	1	0	-4.366843	-3.998676	2.431167
95	7	0	2.603850	2.814638	-2.281832
96	7	0	-2.603850	-2.814638	2.281832
97	6	0	2.027303	3.640581	-1.233325
98	1	0	2.395866	4.695904	-1.299234
99	1	0	2.441330	3.265775	-0.275794
100	6	0	-2.027303	-3.640581	1.233325
101	1	0	-2.441330	-3.265775	0.275794
102	1	0	-2.395866	-4.695904	1.299234
103	6	0	-0.513846	-3.654420	1.157101
104	1	0	-0.082705	-2.637877	1.197720

105	1	0	-0.201607	-4.115377	0.205064
106	1	0	-0.058512	-4.242714	1.971959
107	6	0	0.513846	3.654420	-1.157101
108	1	0	0.058512	4.242714	-1.971959
109	1	0	0.201607	4.115377	-0.205064
110	1	0	0.082705	2.637877	-1.197720
111	6	0	-2.024728	-3.003086	3.606236
112	1	0	-0.947795	-2.774457	3.533762
113	1	0	-2.441427	-2.220311	4.264830
114	6	0	2.024728	3.003086	-3.606236
115	1	0	0.947795	2.774457	-3.533762
116	1	0	2.441427	2.220311	-4.264830
117	6	0	2.234892	4.369767	-4.243279
118	1	0	3.302571	4.571869	-4.433181
119	1	0	1.845640	5.189144	-3.614692
120	1	0	1.717012	4.424834	-5.214170
121	6	0	-2.234892	-4.369767	4.243279
122	1	0	-1.845640	-5.189144	3.614692
123	1	0	-3.302571	-4.571869	4.433181
124	1	0	-1.717012	-4.424834	5.214170
125	6	0	0.081605	3.695415	2.852825
126	1	0	-0.039907	2.984545	3.690923
127	1	0	-0.804662	3.590806	2.202151
128	6	0	-0.081605	-3.695415	-2.852825
129	1	0	0.804662	-3.590806	-2.202151
130	1	0	0.039907	-2.984545	-3.690923
131	6	0	2.228415	4.364896	2.217854
132	1	0	2.183486	5.018375	1.321070
133	1	0	3.229168	3.903410	2.255475
134	6	0	-2.228415	-4.364896	-2.217854
135	1	0	-3.229168	-3.903410	-2.255475
136	1	0	-2.183486	-5.018375	-1.321070
137	6	0	0.326999	5.111276	3.347418
138	1	0	-0.198869	5.326965	4.288860
139	1	0	-0.006243	5.848201	2.595293
140	6	0	1.846478	5.142140	3.459121
141	1	0	2.273016	6.155602	3.482374
142	1	0	2.181939	4.601632	4.362478
143	6	0	-1.846478	-5.142140	-3.459121
144	1	0	-2.181939	-4.601632	-4.362478
145	1	0	-2.273016	-6.155602	-3.482374
146	6	0	-0.326999	-5.111276	-3.347418
147	1	0	0.006243	-5.848201	-2.595293
148	1	0	0.198869	-5.326965	-4.288860
149	8	0	-2.281159	1.578566	-1.993706
150	8	0	2.281159	-1.578566	1.993706
151	6	0	2.123287	-2.071119	3.313223
152	1	0	1.685431	-1.270534	3.930826
153	6	0	-2.123287	2.071119	-3.313223
154	1	0	-1.685431	1.270534	-3.930826
155	6	0	-3.155144	2.506806	-1.355957
156	1	0	-3.558552	2.031197	-0.448740
157	6	0	3.155144	-2.506806	1.355957
158	1	0	3.558552	-2.031197	0.448740
159	1	0	1.413662	-2.925385	3.304406

160	1	0	2.576114	-3.411284	1.073676
161	1	0	-1.413662	2.925385	-3.304406
162	1	0	-2.576114	3.411284	-1.073676
163	6	0	3.516295	-2.529942	3.743523
164	1	0	3.467311	-3.407477	4.405713
165	1	0	4.039003	-1.732988	4.297298
166	6	0	4.220204	-2.820724	2.403864
167	1	0	4.582796	-3.856963	2.324968
168	1	0	5.082933	-2.150528	2.254317
169	6	0	-4.220204	2.820724	-2.403864
170	1	0	-5.082933	2.150528	-2.254317
171	1	0	-4.582796	3.856963	-2.324968
172	6	0	-3.516295	2.529942	-3.743523
173	1	0	-4.039003	1.732988	-4.297298
174	1	0	-3.467311	3.407477	-4.405713
175	8	0	5.680526	-0.664616	0.171888
176	8	0	-5.680526	0.664616	-0.171888
177	6	0	7.064839	-0.963026	-0.074550
178	6	0	-7.064839	0.963026	0.074550
179	1	0	5.794481	1.164476	-0.778927
180	1	0	-5.794481	-1.164476	0.778927
181	6	0	7.588208	-0.337991	-1.364149
182	1	0	8.553845	-0.802459	-1.624199
183	1	0	7.767418	0.746285	-1.271826
184	1	0	6.888318	-0.503510	-2.201234
185	6	0	7.912944	-0.528051	1.113510
186	1	0	8.959533	-0.850164	0.980101
187	1	0	7.527016	-0.973926	2.045771
188	1	0	7.908600	0.567911	1.232776
189	6	0	7.077103	-2.477341	-0.206095
190	1	0	8.102006	-2.851688	-0.360724
191	1	0	6.453315	-2.785659	-1.061403
192	1	0	6.668474	-2.945392	0.704105
193	6	0	-7.077103	2.477341	0.206095
194	1	0	-6.668474	2.945392	-0.704105
195	1	0	-6.453315	2.785659	1.061403
196	1	0	-8.102006	2.851688	0.360724
197	6	0	-7.912944	0.528051	-1.113510
198	1	0	-7.527016	0.973926	-2.045771
199	1	0	-8.959533	0.850164	-0.980101
200	1	0	-7.908600	-0.567911	-1.232776
201	6	0	-7.588208	0.337991	1.364149
202	1	0	-7.767418	-0.746285	1.271826
203	1	0	-8.553845	0.802459	1.624199
204	1	0	-6.888318	0.503510	2.201234
205	6	0	-5.260067	-1.443360	-1.282457
206	6	0	-5.372251	-2.893255	-3.679598
207	6	0	-4.624288	-0.963234	-2.432585
208	6	0	-5.946701	-2.654832	-1.345184
209	6	0	-6.008592	-3.376285	-2.538972
210	6	0	-4.673996	-1.684511	-3.621513
211	1	0	-4.083718	-0.012311	-2.377551
212	1	0	-6.452646	-3.031370	-0.447812
213	1	0	-6.558763	-4.320568	-2.576997
214	1	0	-4.170774	-1.299625	-4.514316

215	1	0	-5.418941	-3.456601	-4.615751
216	6	0	5.260067	1.443360	1.282457
217	6	0	5.372251	2.893255	3.679598
218	6	0	4.624288	0.963234	2.432585
219	6	0	5.946701	2.654832	1.345184
220	6	0	6.008592	3.376285	2.538972
221	6	0	4.673996	1.684511	3.621513
222	1	0	4.083718	0.012311	2.377551
223	1	0	6.452646	3.031370	0.447812
224	1	0	6.558763	4.320568	2.576997
225	1	0	4.170774	1.299625	4.514316
226	1	0	5.418941	3.456601	4.615751

Sum of electronic and thermal Free Energies = -3688.576170 A.U./particle

Monomeric compound (2-mon)

Standard orientation:

Center Number	Atomic Number	Atomic Type	Coordinates (Angstroms) X	Y	Z
1	19	0	2.820408	-0.475521	1.072463
2	5	0	0.666251	1.369316	2.145792
3	1	0	-0.053253	2.144837	2.762145
4	1	0	0.825014	0.309706	2.754089
5	1	0	1.745320	1.903429	1.840479
6	32	0	-0.251487	0.986468	0.306057
7	1	0	-0.072694	2.146344	-0.741639
8	8	0	0.795724	-0.308020	-0.615464
9	6	0	1.058493	-0.338387	-2.008731
10	6	0	1.733249	-1.682740	-2.276000
11	1	0	1.057469	-2.516352	-2.019697
12	1	0	2.647386	-1.775507	-1.660778
13	1	0	2.023841	-1.787626	-3.336185
14	6	0	2.025000	0.781423	-2.389029
15	1	0	2.934945	0.724594	-1.763462
16	1	0	1.565204	1.770596	-2.230605
17	1	0	2.330761	0.709639	-3.447813
18	6	0	-0.215514	-0.233362	-2.838940
19	1	0	-0.917932	-1.045678	-2.588968
20	1	0	0.014075	-0.296215	-3.916294
21	1	0	-0.729845	0.724425	-2.656806
22	8	0	4.803584	-1.586722	-0.386588
23	6	0	-2.187653	0.432798	0.033591
24	6	0	-4.836278	-0.553183	-0.479312
25	6	0	-2.489456	-0.948996	0.045332
26	6	0	-3.270672	1.333645	-0.183832
27	6	0	-4.542389	0.809675	-0.449471
28	6	0	-3.784207	-1.416829	-0.205700
29	1	0	-5.363394	1.501092	-0.642630
30	1	0	-3.947456	-2.498221	-0.183795
31	6	0	-6.258455	-1.025222	-0.772641
32	6	0	-7.213344	-0.463303	0.285508
33	1	0	-7.217231	0.638633	0.295688
34	1	0	-6.926840	-0.806984	1.293875
35	1	0	-8.246463	-0.799973	0.089505
36	6	0	-6.685413	-0.530999	-2.158268
37	1	0	-6.669543	0.568590	-2.227452
38	1	0	-7.712415	-0.865734	-2.386797
39	1	0	-6.014601	-0.926371	-2.939712
40	6	0	-6.375408	-2.546711	-0.755325
41	1	0	-6.112064	-2.967818	0.229389
42	1	0	-5.728275	-3.017140	-1.514385
43	1	0	-7.414683	-2.842925	-0.975074
44	6	0	-3.151373	2.874695	-0.180377
45	6	0	-2.256524	3.378601	0.954688
46	1	0	-2.562045	2.947852	1.923224
47	1	0	-2.333478	4.476520	1.031638
48	1	0	-1.189881	3.161519	0.812959

49	6	0	-2.624808	3.350229	-1.539792
50	1	0	-1.629483	2.939495	-1.765433
51	1	0	-2.549151	4.452358	-1.557963
52	1	0	-3.314116	3.043745	-2.345570
53	6	0	-4.507070	3.562627	0.035088
54	1	0	-5.003325	3.214209	0.956860
55	1	0	-5.203119	3.423355	-0.807058
56	1	0	-4.346127	4.648950	0.130395
57	6	0	-1.457370	-2.022089	0.297195
58	1	0	-0.704653	-2.001096	-0.507649
59	1	0	-1.958344	-3.016961	0.239697
60	7	0	-0.718778	-1.874106	1.539886
61	6	0	0.338260	-2.866815	1.589050
62	1	0	0.981511	-2.675806	0.705269
63	1	0	-0.053377	-3.903393	1.437011
64	6	0	1.168561	-2.837943	2.859070
65	1	0	1.439790	-1.805641	3.140539
66	1	0	2.087744	-3.433992	2.732465
67	1	0	0.631155	-3.263287	3.721314
68	6	0	-1.561814	-1.803285	2.727883
69	1	0	-0.923634	-1.526649	3.584213
70	1	0	-2.241753	-0.944836	2.589566
71	6	0	-2.367942	-3.052602	3.055042
72	1	0	-1.724387	-3.933958	3.220792
73	1	0	-3.079726	-3.306416	2.251952
74	1	0	-2.955719	-2.898883	3.974026
75	6	0	5.062893	-2.985885	-0.548491
76	1	0	4.108976	-3.538536	-0.478995
77	1	0	5.719075	-3.328810	0.274524
78	6	0	5.467713	-0.854332	-1.426544
79	1	0	5.874822	0.072511	-0.994210
80	1	0	4.725661	-0.568819	-2.199208
81	6	0	6.495019	-1.809327	-1.997137
82	1	0	7.398762	-1.831127	-1.363629
83	1	0	6.798529	-1.547149	-3.020766
84	6	0	5.744322	-3.130730	-1.896892
85	1	0	4.989686	-3.197743	-2.700223
86	1	0	6.387230	-4.020508	-1.955744
87	8	0	4.282635	1.706149	0.312138
88	6	0	5.205876	2.011955	1.356360
89	1	0	6.135085	1.440754	1.186160
90	6	0	3.622015	2.909389	-0.113585
91	1	0	3.976738	3.167410	-1.130274
92	1	0	4.782298	1.696829	2.334211
93	1	0	2.535393	2.719707	-0.157324
94	6	0	3.998468	3.968863	0.903501
95	1	0	3.965540	4.986900	0.489816
96	1	0	3.307965	3.921047	1.763351
97	6	0	5.391391	3.516337	1.320591
98	1	0	6.133769	3.790799	0.551007
99	1	0	5.726298	3.928158	2.283524

Sum of electronic and thermal Free Energies = -1844.284211 A.U./particle

Intermediate Int1

Standard orientation:

Center Number	Atomic Number	Atomic Type	Coordinates (Angstroms) X	Y	Z
1	19	0	-0.568853	-2.046102	0.182660
2	5	0	2.042421	-0.525648	0.701333
3	1	0	2.370113	-1.688339	0.926431
4	1	0	1.191379	-0.139703	1.523752
5	1	0	1.646200	-0.409419	-0.459331
6	19	0	0.572164	2.064450	-0.183019
7	5	0	-2.039493	0.543199	-0.701304
8	1	0	-2.368646	1.705983	-0.923981
9	1	0	-1.640546	0.426728	0.458415
10	1	0	-1.189774	0.158577	-1.525712
11	32	0	-3.675670	-0.688563	-1.025165
12	1	0	-3.863130	-1.068852	-2.537710
13	32	0	3.677895	0.705323	1.030556
14	1	0	3.859333	1.088481	2.542772
15	8	0	-3.249030	-2.412125	-0.381917
16	8	0	3.254547	2.428282	0.383228
17	6	0	3.645730	3.653656	0.985662
18	6	0	-3.639774	-3.637176	-0.985161
19	6	0	-3.411266	-4.716766	0.068478
20	1	0	-4.069474	-4.562781	0.940079
21	1	0	-2.365663	-4.688080	0.424264
22	1	0	-3.606031	-5.724580	-0.337119
23	6	0	3.420185	4.732357	-0.069530
24	1	0	3.615453	5.740353	0.335356
25	1	0	2.375114	4.704530	-0.427012
26	1	0	4.079691	4.576727	-0.939841
27	6	0	5.112599	3.659807	1.403766
28	1	0	5.396262	4.649597	1.799806
29	1	0	5.769087	3.431362	0.547731
30	1	0	5.310055	2.913112	2.189779
31	6	0	2.752669	3.940959	2.189335
32	1	0	3.019852	4.895103	2.676988
33	1	0	2.838763	3.135378	2.938106
34	1	0	1.695820	4.003845	1.872106
35	6	0	-2.747956	-3.921851	-2.190366
36	1	0	-1.690752	-3.984703	-1.874289
37	1	0	-2.835380	-3.114855	-2.937479
38	1	0	-3.015069	-4.875241	-2.679542
39	6	0	-5.107244	-3.644962	-1.401277
40	1	0	-5.763043	-3.418006	-0.544309
41	1	0	-5.389927	-4.634877	-1.797716
42	1	0	-5.306779	-2.897969	-2.186441
43	8	0	-0.553929	4.326824	-1.285363
44	8	0	0.556842	-4.311446	1.281720
45	6	0	-5.579621	-0.302715	-0.441284
46	6	0	-8.297635	-0.078608	0.454064
47	6	0	-5.996589	-0.790099	0.818348
48	6	0	-6.549273	0.383769	-1.227493

49	6	0	-7.869882	0.448518	-0.764361
50	6	0	-7.323243	-0.672315	1.246053
51	1	0	-8.618270	0.944012	-1.384241
52	1	0	-7.577573	-1.068850	2.233580
53	6	0	5.580982	0.306952	0.451150
54	6	0	8.288758	0.024978	-0.459840
55	6	0	6.000997	0.784085	-0.811452
56	6	0	6.542770	-0.392430	1.236160
57	6	0	7.858065	-0.488471	0.763353
58	6	0	7.322931	0.640130	-1.245767
59	1	0	8.599822	-0.999013	1.378719
60	1	0	7.580046	1.031184	-2.234721
61	6	0	-9.761310	0.043511	0.871319
62	6	0	9.744714	-0.137850	-0.890050
63	6	0	10.083314	-1.628781	-0.990508
64	1	0	11.132836	-1.765539	-1.304930
65	1	0	9.436187	-2.128183	-1.731538
66	1	0	9.952138	-2.147732	-0.027075
67	6	0	10.019145	0.503529	-2.247362
68	1	0	9.811948	1.586688	-2.238665
69	1	0	9.416298	0.044939	-3.048874
70	1	0	11.080667	0.370069	-2.514548
71	6	0	10.662183	0.524560	0.142361
72	1	0	10.438823	1.601059	0.232515
73	1	0	11.719117	0.418075	-0.158274
74	1	0	10.553414	0.074434	1.142153
75	6	0	-10.149508	1.523367	0.953982
76	1	0	-10.029042	2.037222	-0.013495
77	1	0	-9.524926	2.051333	1.694547
78	1	0	-11.205306	1.628156	1.259522
79	6	0	-10.647107	-0.659098	-0.162342
80	1	0	-10.542142	-0.216592	-1.165944
81	1	0	-11.709937	-0.583465	0.126752
82	1	0	-10.388316	-1.728795	-0.238248
83	6	0	-10.026780	-0.592849	2.232755
84	1	0	-9.444220	-0.108531	3.034182
85	1	0	-9.787410	-1.669359	2.236525
86	1	0	-11.093941	-0.488931	2.490495
87	6	0	-6.265885	1.063522	-2.586682
88	6	0	6.256139	-1.051503	2.604919
89	6	0	-4.914953	1.784952	-2.596948
90	1	0	-4.806892	2.438629	-1.713474
91	1	0	-4.834485	2.414332	-3.500333
92	1	0	-4.049977	1.108506	-2.621915
93	6	0	-6.328598	0.020260	-3.708323
94	1	0	-5.583718	-0.777914	-3.571574
95	1	0	-6.140863	0.496549	-4.687422
96	1	0	-7.328679	-0.445116	-3.743335
97	6	0	-7.306364	2.142725	-2.920096
98	1	0	-7.392130	2.893103	-2.115483
99	1	0	-8.309194	1.732990	-3.117649
100	1	0	-6.998218	2.667178	-3.839570
101	6	0	7.290661	-2.130488	2.956445
102	1	0	8.296128	-1.722688	3.144487
103	1	0	7.370470	-2.896713	2.166304

104	1	0	6.980933	-2.635517	3.886154
105	6	0	4.901757	-1.766363	2.624879
106	1	0	4.816511	-2.379815	3.538737
107	1	0	4.793284	-2.434462	1.752382
108	1	0	4.039691	-1.085954	2.635679
109	6	0	6.324630	0.008151	3.710814
110	1	0	7.327848	0.467184	3.739523
111	1	0	6.133037	-0.452137	4.696791
112	1	0	5.585484	0.809290	3.561827
113	6	0	5.074220	1.482430	-1.776887
114	1	0	5.635640	1.701094	-2.715425
115	1	0	4.769918	2.457383	-1.364092
116	6	0	-5.063048	-1.476789	1.785436
117	1	0	-4.755920	-2.452918	1.377281
118	1	0	-5.620586	-1.692925	2.726882
119	7	0	3.845335	0.759832	-2.046740
120	7	0	-3.836366	-0.748310	2.048234
121	6	0	2.955266	1.589975	-2.842911
122	1	0	3.465498	1.977513	-3.759362
123	1	0	2.742000	2.488431	-2.230141
124	6	0	-2.942596	-1.572688	2.846571
125	1	0	-2.727796	-2.472826	2.236718
126	1	0	-3.450921	-1.958653	3.764713
127	6	0	-1.645258	-0.896632	3.247504
128	1	0	-1.181742	-0.368506	2.397148
129	1	0	-0.925791	-1.641622	3.625905
130	1	0	-1.785790	-0.151818	4.048699
131	6	0	1.656348	0.918774	-3.247230
132	1	0	1.795542	0.174855	-4.049491
133	1	0	0.940059	1.666762	-3.625751
134	1	0	1.189521	0.390671	-2.398610
135	6	0	-4.048572	0.600411	2.551917
136	1	0	-3.065631	1.101535	2.577845
137	1	0	-4.633228	1.148168	1.790000
138	6	0	4.053598	-0.587399	-2.555766
139	1	0	3.069477	-1.086176	-2.581844
140	1	0	4.638328	-1.139324	-1.796993
141	6	0	4.737896	-0.698523	-3.911705
142	1	0	5.753892	-0.270631	-3.895012
143	1	0	4.171277	-0.184140	-4.707490
144	1	0	4.838665	-1.755205	-4.210704
145	6	0	-4.735945	0.714727	3.905955
146	1	0	-4.171520	0.201489	4.704065
147	1	0	-5.752201	0.287454	3.887265
148	1	0	-4.836742	1.772060	4.202587
149	6	0	-1.746541	4.231959	-2.074165
150	1	0	-2.597686	3.964203	-1.416438
151	1	0	-1.625021	3.413389	-2.802592
152	6	0	1.750827	-4.217101	2.068379
153	1	0	1.630893	-3.398563	2.797133
154	1	0	2.600701	-3.949742	1.408894
155	6	0	-0.214451	5.701687	-1.093393
156	1	0	0.691787	5.940118	-1.684129
157	1	0	0.025603	5.864818	-0.027286
158	6	0	0.217901	-5.686250	1.088275

159	1	0	-0.022557	-5.848351	0.022091
160	1	0	-0.688022	-5.925669	1.679077
161	6	0	-1.934789	5.602221	-2.693050
162	1	0	-2.978871	5.802213	-2.974322
163	1	0	-1.308292	5.708835	-3.595605
164	6	0	-1.411273	6.499591	-1.579376
165	1	0	-1.141418	7.513660	-1.907764
166	1	0	-2.166504	6.589513	-0.778583
167	6	0	1.415246	-6.484347	1.572790
168	1	0	2.169761	-6.573862	0.771275
169	1	0	1.145707	-7.498603	1.900865
170	6	0	1.939828	-5.587580	2.686453
171	1	0	1.314266	-5.694624	3.589604
172	1	0	2.984230	-5.787616	2.966508
173	8	0	0.656011	-3.096881	-2.001627
174	8	0	-0.651214	3.118691	2.000342
175	6	0	-1.948268	3.656052	1.718541
176	1	0	-1.844469	4.453235	0.961849
177	6	0	1.952279	-3.636085	-1.719936
178	1	0	1.847357	-4.433388	-0.963508
179	6	0	0.438958	-3.051717	-3.411086
180	1	0	-0.240457	-3.876193	-3.707297
181	6	0	-0.434803	3.072949	3.409912
182	1	0	0.243177	3.898336	3.706941
183	1	0	-2.572804	2.855674	1.276091
184	1	0	0.065665	2.120829	3.656407
185	1	0	2.577720	-2.836682	-1.277119
186	1	0	-0.060041	-2.098901	-3.657857
187	6	0	-2.498096	4.150634	3.047049
188	1	0	-3.596783	4.102108	3.092354
189	1	0	-2.199650	5.198607	3.223638
190	6	0	-1.800560	3.236643	4.048714
191	1	0	-2.317093	2.263961	4.116902
192	1	0	-1.744886	3.658676	5.062962
193	6	0	1.804113	-3.217700	-4.050614
194	1	0	1.747206	-3.640939	-5.064291
195	1	0	2.321497	-2.245645	-4.120565
196	6	0	2.501529	-4.131326	-3.048507
197	1	0	2.202828	-5.179305	-3.224528
198	1	0	3.600224	-4.083127	-3.094144

Sum of electronic and thermal Free Energies = -4379.108833 A.U./particle

References

[S1] M. J. Frisch, G. W. Trucks, H. B. Schlegel, G. E. Scuseria, M. A. Robb, J. R. Cheeseman, G. Scalmani, V. Barone, G. A. Petersson, H. Nakatsuji, X. Li, M. Caricato, A. V. Marenich, J. Bloino, B. G. Janesko, R. Gomperts, B. Mennucci, H. P. Hratchian, J. V. Ortiz, A. F. Izmaylov, J. L. Sonnenberg, D. Williams-Young, F. Ding, F. Lipparini, F. Egidi, J. Goings, B. Peng, A. Petrone, T. Henderson, D. Ranasinghe, V. G. Zakrzewski, J. Gao, N. Rega, G. Zheng, W. Liang, M. Hada, M. Ehara, K. Toyota, R. Fukuda, J. Hasegawa, M. Ishida, T. Nakajima, Y. Honda, O. Kitao, H. Nakai, T. Vreven, K. Throssell, J. A. Montgomery, Jr., J. E. Peralta, F. Ogliaro, M. J. Bearpark, J. J. Heyd, E. N. Brothers, K. N. Kudin, V. N. Staroverov, T. A. Keith, R. Kobayashi, J. Normand, K. Raghavachari, A. P. Rendell, J. C. Burant, S. S. Iyengar, J. Tomasi, M. Cossi, J. M. Millam, M. Klene, C. Adamo, R. Cammi, J. W. Ochterski, R. L. Martin, K. Morokuma, O. Farkas, J. B. Foresman, D. J. Fox, Gaussian 16 (Revision A.03), Gaussian Inc., Wallingford CT, 2016.

[S2] Y. Zhao, D. G. Truhlar, Theor. Chem. Acc. 2008, 120, 215–241.

[S3] A. V. Marenich, C. J. Cramer, D. G. Truhlar, J. Phys. Chem. B 2009, 113, 6378–6396

[S4] a) T. H. Dunning, Jr. J. Chem. Phys. 1989, 90, 1007–1023; b) B. P. Prascher, D. E. Woon, K. A. Peterson, T. H. Dunning, Jr., A. K. Theor. Chem. Acc. 2011, 128, 69–82.

[S5] a) B. Metz, H. Stoll, M. Dolg, J. Chem. Phys. 2000, 113, 2563–2569; b) K. A. Peterson, J. Chem. Phys. 2003, 119, 11099–11112.

[S6] P. Hay, W.R. Wadt, J. Chem. Phys. 1985, 82, 299-31.
